# Causal relationships between rheumatism and dyslipidemia: A two-sample Mendelian randomization study

**DOI:** 10.3389/fendo.2022.961505

**Published:** 2022-08-31

**Authors:** Guangyang Zhang, Yuanqing Cai, Jialin Liang, Jianan Zhang, Zhaopu Jing, Leifeng Lv, Rupeng Zhang, Jidong Song, Xiaoqian Dang, Qichun Song

**Affiliations:** ^1^ Department of Orthopedics, The Second Affiliated Hospital of Xi’an Jiaotong University, Xi’an, China; ^2^ Department of Orthopedics, Xi ‘an Jiaotong University Health Science Center, Xi’an, China

**Keywords:** ankylosing spondylitis, rheumatoid arthritis, systemic lupus erythematosus, dyslipidemia, Mendelian randomization

## Abstract

**Background:**

Dyslipidemia is often observed in rheumatic diseases, such as ankylosing spondylitis (AS), rheumatoid arthritis (RA), and systemic lupus erythematosus (SLE), yet it remains to be detected whether rheumatic diseases have a causal effect on dyslipidemia.

**Methods:**

Significant (*P* < 5 × 10^-8^) and independent (r^2^ < 0.1) single-nucleotide polymorphisms in genome-wide association studies were selected as instrumental variables to conduct Mendelian randomization (MR) analysis. Inverse variance weighted, weighted median, and MR–Egger regression were adopted for the causal inference. Subsequently, sensitivity analysis was conducted to assess the stability and reliability of MR.

**Results:**

The MR results revealed positive causal relationships of AS with total cholesterol (TC) (β = 0.089, 95% CI = 0.050 to 0.128, *P* = 6.07 × 10^-6^), low-density lipoprotein (LDL) (β = 0.087, 95% CI = 0.047 to 0.127, *P* = 1.91 × 10^-5^), and high-density lipoprotein (HDL) (β = 0.043, 95% CI = 0.001 to 0.074, *P* = 0.009). There was no causal effect of RA on TC (β = 0.008, 95% CI = 4.86 × 10^-4^ to 0.017, *P* = 0.064), LDL (β = 6.4 × 10^-4^, 95% CI = -0.008 to 0.007, *P* = 0.871), or HDL (β = 0.005, 95% CI = -0.003 to 0.013, *P* = 0.200). Additionally, SLE had negative causal links for TC (β = -0.025, 95% CI = -0.036 to -0.015, *P* = 4.42 × 10^-6^), LDL (β = -0.015, 95% CI = -0.025 to -0.005, *P* = 0.003), and HDL (β = -0.013, 95% CI = -0.021 to -0.004, *P* = 0.004). The results were stable and reliable.

**Conclusion:**

This study suggested positive causal effects of AS on TC, LDL, and HDL and negative causal effects of SLE on these cholesterol levels, which could provide much help for the pathogenesis and treatment of rheumatic disease patients with dyslipidemia.

## Introduction

Rheumatic diseases are rare heterogeneous disorders associated with substantial morbidity and mortality, and these include ankylosing spondylitis (AS), rheumatoid arthritis (RA), and systemic lupus erythematosus (SLE) (1). AS is a chronic inflammatory disease that mainly influences the sacroiliac joints and spine and covers people with both non-radiographic and radiographic axial spondyloarthritis. This disease usually attacks individuals over 20 years old with a male-to-female ratio of 2:1 (2). Nearly 0.32%–1.4% of the population is affected by AS, which has higher mortality rates than normal individuals (3, 4). RA is one of the most common forms of chronic arthritis, affecting nearly 1% of the world’s population and placing a substantial burden on both the individual and society (5). SLE is an autoimmune disease in which healthy cells or tissues are attacked by the immune system, causing inflammation of multiple organs, including the kidneys, skin, cardiovascular system, and central nervous system (6). The standardized mortality for SLE is estimated at 2.4%–5.9% despite advances in treatment (7). All three of these rheumatic diseases have burdened both individuals and society.

Dyslipidemia refers to the abnormality of lipid metabolism, one of the most important risk factors for atherosclerosis in these rheumatic diseases, contributing to the increased mortality of AS, RA, and SLE (8, 9). High-density lipoprotein (HDL) provides a protective effect against atherosclerosis, while low-density lipoprotein (LDL) and total cholesterol (TC) have an adverse influence (10). Some scholars found that lower HDL was surprisingly associated with an increased risk of complications in patients with AS (11, 12). Furthermore, Kucuk et al. (13) indicated that the LDL/HDL ratio was higher in AS patients. Regarding RA, Boyer et al. (14) indicated that lower HDL cholesterol levels were more prevalent in RA patients and could cause increased morbidity and mortality. However, the cholesterol levels in RA may be controversial. According to a previous comparative study, individuals with RA had lower TC and LDL but higher HDL levels than those of the general population (15). Regarding dyslipidemia in SLE, it was indicated that 73.4% of SLE patients have dyslipidemia (16). Similarly, a cohort study determined that 36.3% of participants had dyslipidemia (higher TC and LDL, lower HDL) at the time of SLE diagnosis, while it rose to over 60% 3 years later (17). Nevertheless, it is uncertain whether AS, RA, and SLE have causal effects on abnormal lipid metabolism, meaning that more research is needed to determine their potential relationships.

Mendelian randomization (MR) is a powerful method that is widely used in causal inference by taking genetic variants as instrumental variables (IVs) and abiding by the law of independent assortment (18, 19). The MR method could minimize the confounders to a large extent and avoid reverse causality because genotypes appear before the occurrence of disease and are largely unrelated to lifestyle or environmental factors after birth (20). Hence, this study took single-nucleotide polymorphisms (SNPs) as IVs to perform a two-sample MR analysis to estimate the causal effect of rheumatic diseases (AS, RA, and SLE) on dyslipidemia (TC, LDL, and HDL).

## Materials and methods

### Study design

**Figure 1:**
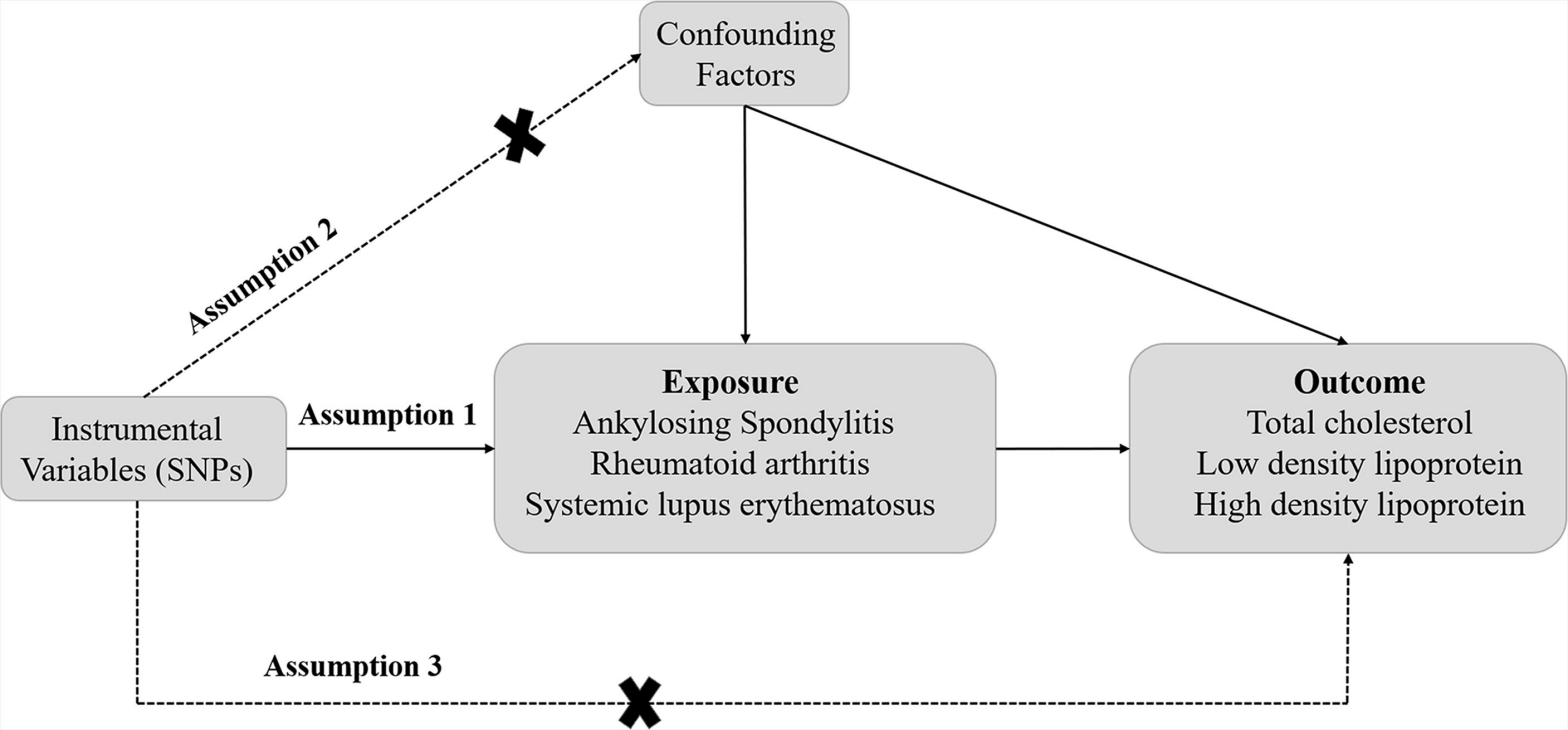
The schematic diagram of Mendelian randomization (MR). Three assumptions should be met, as follows: Assumption 1: The SNPs should be closely related to exposures; Assumption 2: The IVs selected are supposed to be independent of confounders; Assumption 3: SNPs should influence the outcomes just through the exposure. (IVs, instrumental variables; SNPs, single-nucleotide polymorphisms).

The design of this study is displayed in **Figure 1**. A two-sample MR analysis was conducted to explore the causal effect of rheumatic diseases on dyslipidemia. Three assumptions need to be met when performing MR analysis. First, the SNPs should be closely related to exposures. Second, the IVs selected are supposed to be independent of confounders. Third, SNPs should influence the outcomes just through exposure (19). In this study, additional ethical approval or informed consent was not required because the data used came from publicly published data.

### Data sources

The summary-level genetic data used in this two-sample MR analysis originated from genome-wide association study (GWAS) datasets. Genetic variants for AS were obtained from a GWAS including 22,647 individuals (9,069 AS patients and 13,578 controls) and 99,962 SNPs of European ancestry (21). The selection of RA-associated genetic predictors was based on a GWAS of 58,284 objects (14,361 RA cases and 43,923 controls) and 8,747,963 SNPs of European descent (22). Additionally, there were 5,201 SLE patients and 9,066 controls (altogether 14,267 individuals) of European ancestry with 7,071,163 SNPs selected as the genetic variants from another GWAS (23). Moreover, one GWAS with 94,595 subjects of European descent from 23 studies was used to identify IVs for TC, LDL, and HDL levels, the details of which have been elucidated by previous research (24). All of the data adopted in this current study are publicly available at the GWAS summary datasets (https://gwas.mrcieu.ac.uk).

### Selection of instrumental variables

This two-sample MR analysis took the genome-wide significant (*P* < 5 × 10^-8^) SNPs as IVs to assess the causal effects of AS, RA, and SLE on TC, LDL, and HDL. The clumping process (r^2^ < 0.1, window size = 10,000 kb) was performed with the European samples from the 1000 Genomes Project to wipe off the influence of linkage disequilibrium. Then, the Steiger test was also adopted to remove those SNPs that explain more of the variance in the dyslipidemia than in rheumatic diseases. Moreover, the F-statistics were calculated, and the sensitive SNPs were extracted for further analysis based on F-statistics ≥10.

### Statistical analyses

This study adopted the inverse variance weighted (IVW), weighted median, and MR–Egger methods to explore the causal relationships between AS, RA, and SLE and TC, LDL, and HDL. The IVW method analyzes each Wald ratio and provides a consistent estimate of the causal effect when all IVs are valid, which is primarily used in the results with no heterogeneity or directional pleiotropy (25). The weighted median method gives unbiased estimates even when up to 50% of the information comes from invalid IVs (26). It would provide a more accurate estimation than IVW and MR–Egger when heterogeneity exists in the results. MR–Egger regression was conducted to test horizontal pleiotropy. However, the effect size rather than the statistical significance of MR–Egger was concentrated because the statistical power is low (27).

### Sensitivity analyses

Additionally, heterogeneity was assessed by Cochran’s Q test and I^2^ statistics. Generally, there was no heterogeneity in the results when I^2^ < 50%. MR–Egger regression was used to detect pleiotropy. Moreover, this study conducted a sensitivity analysis using the “leave-one-out” sensitivity test to evaluate the reliability and stability of MR results. All statistical analyses were performed with the “Two-Sample MR” package in R (version 4.1.2) software. The results were statistically significant when *P* < 0.05.

## Results

### The single-nucleotide polymorphisms selected for this study

This study chose rheumatic disease (AS, RA, and SLE) as exposures to conduct a two-sample MR analysis for dyslipidemia. Significant and independent (*P* < 5 × 10^-8^, r^2^ < 0.1) SNPs were extracted, and the remaining SNPs were chosen for further MR analysis after excluding the weak IVs (F < 10). **Supplementary Tables S1–S9** show the basic information of the selected SNPs for MR analysis. For AS exposure, there were 63 AS-related SNPs with a mean value of F = 178.32 for TC, 63 SNPs with a mean of F = 178.32 for LDL, and 62 SNPs with a mean value of F = 177.85 for HDL selected for MR analysis. For RA exposure, 78, 79, and 79 SNPs were selected for analysis, with mean F values of 160.65, 159.74, and 159.74 for TC, LDL, and HDL, respectively. For SLE exposure, 49 SNPs for TC, LDL, and HDL with the same mean of F = 83.89 were selected for MR analysis.

### Causal effect of ankylosing spondylitis on dyslipidemia

**Figure 2:**
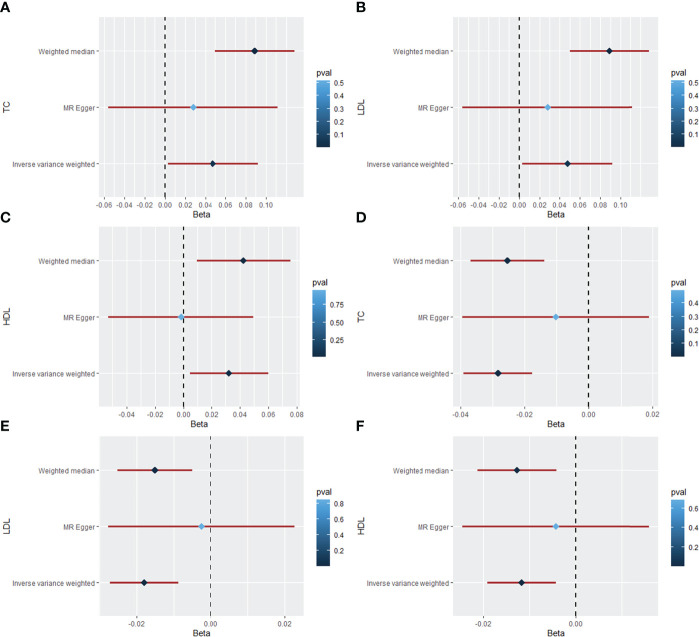
The forest plot for causal effects of rheumatic diseases on dyslipidemia. **(A)** Forest plot of the casual effect of AS on TC. **(B)** Forest plot of the casual effect of AS on LDL. **(C)** Forest plot of the casual effect of AS on HDL. **(D)** Forest plot of the casual effect of SLE on TC. **(E)** Forest plot of the casual effect of SLE on LDL. **(F)** Forest plot of the casual effect of SLE on HDL. (AS, ankylosing spondylitis; RA, rheumatoid arthritis; SLE, systemic lupus erythematosus; TC, total cholesterol; LDL, low-density lipoprotein; HDL, high-density lipoprotein; beta (β), a ratio of changes in standard deviations).

The MR results of AS on TC are listed in **Table 1**, **Figure 2A**, and **Supplementary Figure S1**, which were reported as beta (β). According to Cochran’s Q, I^2^, and MR–Egger regression tests, there was heterogeneity (Q = 375.20, *P* = 9.49 × 10^-47^; I^2^ = 83.74%) but no pleiotropy (intercept = 0.002, *P* = 0.589) in the results. Therefore, the weighted median method was adopted for the causal relationship between AS and dyslipidemia (28). The weighted median method showed that AS had a positive causal effect on TC (β = 0.089, 95% CI = 0.050 to 0.128, *P* = 6.07 × 10^-6^), and a similar result was obtained from IVW (β = 0.048, 95% CI = 0.003 to 0.092, *P* = 0.035). Then, the “leave-one-out” sensitivity indicated that the causal relationship between AS and TC was not affected by individual SNPs (**Supplementary Figure S1D**), meaning that the result was stable and reliable. Additionally, **Table 1**, **Figure 2B**, and **Supplementary Figure S2** show the MR results of AS on LDL. According to the weighted median method, AS was positively associated with higher LDL levels (β = 0.087, 95% CI = 0.047 to 0.127, *P* = 1.91 × 10^-5^). The IVW method showed a similar result (β = 0.041, 95% CI = 0.003 to 0.079, *P* = 0.035). Cochran’s Q and I^2^ statistics indicated the existence of heterogeneity (Q = 258.74, *P* = 3.49 × 10^-26^; I^2^ = 76.42%). There was no evidence for directional pleiotropy in the MR–Egger regression analysis (intercept = 0.002, *P* = 0.581). In the “leave-one-out” analysis, no single SNP strongly drove the overall effect of AS on LDL (**Supplementary Figure S2D**). For the HDL outcome, the MR results are listed in **Table 1**, **Figure 2C**, and **Supplementary Figure S3**. There was a positive causal effect of AS on HDL (β = 0.043, 95% CI = 0.001 to 0.074, *P* = 0.009) according to the weighted median method. The association was consistent with the IVW method (β = 0.032, 95% CI = 0.004 to 0.060, *P* = 0.023). Heterogeneity (Q = 156.09, *P* = 1.71 × 10^-10^; I^2^ = 61.56%) but not directional pleiotropy (intercept = 0.003, *P* = 0.130) existed in the MR results, meaning that it was more appropriate to adopt the weighted median method to analyze the results. Then, the “leave-one-out” sensitivity indicated that the causal effect of AS on HDL was not affected by individual SNPs (**Supplementary Figure S3D**).

**Table 1:** The Mendelian randomization (MR) analysis results with regard to causal effect of AS on TC, LDL, and HDL levels.

Outcome	Method	SNP (n)	β	95% CI	*P*-value
TC	Weighted median	63	0.089	0.050, 0.128	6.07 × 10^-6^
	Inverse variance weighted	63	0.048	0.003, 0.092	0.035
	MR Egger	63	0.028	-0.056, 0.112	0.515
LDL	Weighted median	63	0.087	0.047, 0.127	1.91 × 10^-5^
	Inverse variance weighted	63	0.041	0.003, 0.079	0.035
	MR Egger	63	0.024	-0.048, 0.096	0.522
HDL	Weighted median	62	0.043	0.011, 0.074	0.009
	Inverse variance weighted	62	0.032	0.004, 0.060	0.023
	MR Egger	62	-0.002	-0.053, 0.050	0.998

beta (β), a ratio of changes in standard deviations; AS, ankylosing spondylitis; TC, total cholesterol; LDL, low-density lipoprotein; HDL, high-density lipoprotein; SNP, single-nucleotide polymorphism; CI, confidence interval.

### Causal effect of rheumatoid arthritis on dyslipidemia

In terms of the relationships of RA with TC, LDL, and HDL, the results provided sufficient statistical power for causal analysis between them according to the results in **Supplementary Table S10** and **Supplementary Figures S4–S6**. Nevertheless, none of the three methods provided evidence for a causal relationship between RA and TC (weighted median: β = 0.008, 95% CI = 4.86×10^-4^ to 0.017, *P* = 0.064; IVW: β = 0.004, 95% CI = -0.005 to 0.014, *P* = 0.386; MR–Egger: β = -0.003, 95% CI = -0.009 to 0.014, *P* = 0.764) based on the results in **Supplementary Table S10**. The MR analysis with regard to the causal effect of RA on LDL also showed that there was a null effect between them (weighted median: β = 6.4 × 10^-4^, 95% CI = -0.008 to 0.007, *P* = 0.871; IVW: β = 0.002, 95% CI = -0.006 to 0.009, *P* = 0.635; MR–Egger: β = -0.004, 95% CI = -0.017 to 0.009, *P* = 0.547). Similarly, there was no evidence for the causal effect of RA on HDL according to the weighted median method (β = 0.005, 95% CI = -0.003 to 0.013, *P* = 0.200), IVW (β = 0.006, 95% CI = -7.5 × 10^-4^ to 0.013, *P* = 0.081), and MR–Egger (β = 0.009, 95% CI = -0.002 to 0.021, *P* = 0.124). Hence, there was no causal effect of RA on TC, LDL, and HDL.

### Causal effect of systemic lupus erythematosus on dyslipidemia

**Table 2**, **Figures 2D–F**, and **Supplementary Figures S7–S9** show the MR results of SLE on TC, LDL, and HDL. According to the weighted median method, SLE was negatively associated with LDL (β = 0.025, 95% CI = -0.036 to -0.015, *P* = 4.42 × 10^-6^). The association was consistent with the IVW method (β = -0.028, 95% CI = -0.039 to -0.018, *P* = 2.16 × 10^-7^). According to Cochran’s Q, I^2^, and MR–Egger regression, there was heterogeneity (Q = 153.30, *P* = 7.09 × 10^-15^; I^2^ = 73.26%) but no pleiotropy (intercept = -0.006, *P* = 0.200) in the results, which means that the weighted median method was more appropriate for analysis of the results. The “leave-one-out” sensitivity showed that the causal effect of SLE on TC was not affected by individual SNPs (**Supplementary Figure S7D**). For the LDL outcome, the weighted median (β = -0.015, 95% CI = -0.025 to -0.005, *P* = 0.003) and IVW (β = -0.018, 95% CI = -0.027 to -0.009, *P* = 1.39×10^-4^) methods indicated a negative causal effect of SLE on LDL. Heterogeneity (Q = 108.19, *P* = 5.72 × 10^-8^; I^2^ = 62.11%) but not pleiotropy (intercept = -0.005, *P* = 0.201) existed in the results. The “leave-one-out” analysis showed that the result was reliable and stable (**Supplementary Figure S8D**). Additionally, there was a negative causal effect of SLE on HDL by the weighted median method (β = 0.013, 95% CI = -0.021 to -0.004, *P* = 0.004). The IVW showed a similar result regarding the causal effect of SLE on HDL (β = -0.012, 95% CI = -0.019 to -0.004, *P* = 0.002). There was heterogeneity (Q = 82.86, *P* = 1.18 × 10^-4^; I^2^ = 50.52%) but no pleiotropy (intercept = -0.003, *P* = 0.446) in the results according to Cochran’s Q, I^2^, and MR–Egger regression. The result was not affected by a single SNP according to “leave-one-out” analysis (**Supplementary Figure S9D**).

**Table 2:** The Mendelian randomization (MR) analysis results with regard to causal effect of SLE on TC, LDL, and HDL levels.

Outcome	Method	SNP (n)	β	95% CI	*P*-value
TC	Weighted median	49	-0.025	-0.036, -0.015	4.42 × 10^-6^
	Inverse variance weighted	49	-0.028	-0.039, -0.018	2.16 × 10^-7^
	MR Egger	49	-0.010	-0.039, -0.019	0.494
LDL	Weighted median	49	-0.015	-0.025, -0.005	0.003
	Inverse variance weighted	49	-0.018	-0.027, -0.009	1.39 × 10^-4^
	MR Egger	49	-0.002	-0.028, 0.023	0.848
HDL	Weighted median	49	-0.013	-0.021, -0.004	0.004
	Inverse variance weighted	49	-0.012	-0.019, -0.004	0.002
	MR Egger	49	-0.004	-0.025, 0.016	0.683

beta (β), a ratio of changes in standard deviations; SLE, systemic lupus erythematosus; TC, total cholesterol; LDL, low-density lipoprotein; HDL, high-density lipoprotein; SNP, single-nucleotide polymorphism; CI, confidence interval.

## Discussion

The aim of this study was to explore the relationship between autoimmune rheumatic diseases and dyslipidemia using MR. To the best of our knowledge, this was the first study to assay the causal effect of AS, RA, and SLE on TC, LDL, and HDL based on GWAS datasets. According to the MR results, this study provided strong genetic evidence that AS had a positive causal effect on TC, LDL, and HDL, while SLE had a negative causal effect on TC, LDL, and HDL. However, there were no causal effects of RA on TC, LDL, and HDL according to the MR results. These findings would contribute greatly to research on the mechanism and treatment of dyslipidemia in different kinds of rheumatic diseases.

AS is an inflammatory disease associated with dyslipidemia that involves the spine, peripheral joints, and entheses (29). García-Gómez et al. (30) reported that 20.7% (95% CI: 16.91 to 24.82) of patients with AS had hyperlipidemia in a 10-year prospective study. Additionally, another study found that the LDL/HDL ratio was higher in AS patients than that in controls (13). The conclusions of these observational studies were consistent with our results that AS could increase the levels of LDL. However, low HDL cholesterol levels were also observed in AS individuals (31). It was elucidated that increased disease activity and inflammatory indicators, such as erythrocyte sedimentation rate and C-reactive protein, in patients with AS were related to a decrease in lipids (32). Moreover, AS-related inflammation could increase the levels of HDL-associated serum amyloid A and reduce plasma paraoxonase-1 activity in patients with active AS, which would greatly affect the levels and function of HDL (33). These observational studies could determine the associations but not the causal relationship between HDL and AS due to the existence of confounding factors and reverse causality. Our study indicated a positive causal effect of AS on TC, LDL, and HDL, providing much help for the mechanism and treatment of dyslipidemia in AS patients to prevent complications due to lipid abnormalities. More studies are needed to reveal the potential pathogenesis for the causal relationships between AS and dyslipidemia.

RA is the most common form of chronic arthritis and is associated with abnormal levels of cholesterol (34). Boyer et al. (14) indicated that lower HDL cholesterol levels appeared more prevalent in RA patients and could contribute to the increased morbidity and mortality of RA. In a retrospective study, 79 patients who later developed RA had higher TC and lower HDL cholesterol levels than those in controls (35). However, a 10-year cohort study found that lipid changes commenced prior to RA diagnosis (36). In another retrospective study, RA patients displayed lower mean TC (by 10%) and LDL (by 17%) but no significant difference in HDL levels when compared with those in non-RA controls in the 5 years preceding diagnosis (34), revealing that dyslipidemia and RA might have a similar pathogenesis. RA-related inflammation plays a crucial role in lipid metabolism alterations, which could affect the levels of cholesterol (37). Semb et al. (38) found that lipid levels were initially low and then increased after disease activity was controlled in RA patients with a high inflammatory burden. In our study, the conclusion that RA had no causal effect on TC, LDL, and HDL was different from the results of observational studies, which might be explained by the fact that severe inflammation in RA could change the function and serum levels of lipids (39). The sample sizes, different races, confounders not yet considered and treatment effects might also affect the results regarding the relationship between RA and dyslipidemia. As a result, it remains to be explored whether treatment of RA would improve the abnormal lipid levels in RA patients, as the relationship between them is unclear.

SLE is a chronic autoimmune disease associated with dyslipidemia (40). A previous study reported that prior to treatment with rituximab, 69% of SLE patients had dyslipidemia, among which the major abnormalities were LDL and HDL (41). Elfving et al. (42) showed that 90% of patients with SLE used drugs at the onset of the disease to control the major comorbidities of dyslipidemia, which implied that dyslipidemia in SLE is not caused by medications. Another study also illustrated that the levels of HDL decreased in SLE patients (43), which was consistent with our results. Autoimmunity, inflammation, and oxidative stress in the process of SLE could alter lipid and lipoprotein metabolism (44). Resolvin D1 is a product of the metabolism of the omega-3 polyunsaturated fatty acid docosahexaenoic acid and can improve homeostasis in inflamed tissues. It was found that the levels of resolvin D1 decreased significantly in SLE patients (45). Moreover, Atta et al. (16) showed a positive correlation between Interleukin-6 (IL-6) and C-reaction protein (CRP) levels in a group of patients with dyslipidemia. As a result, it was hypothesized that SLE might cause dyslipidemia by decreasing the levels of resolvin D1 to increase the levels of inflammatory biomarkers such as IL-6 and CRP. This study revealed that it was important to treat SLE when curing dyslipidemia in SLE patients. However, more work is needed to determine the potential mechanism of the causal effect of SLE on dyslipidemia.

Regarding the associations between autoimmune diseases and dyslipidemia, this was the first study to explore their causal relationships with MR analysis, which minimized the unmeasured confounders and reverse causality biases and detected the causality with high precision. Nevertheless, limitations existed in this study. First, whether similar results could be obtained in other ancestries is unknown because this MR analysis focused on individuals of European ancestry. Second, this MR analysis mainly concentrated on the levels of TC, LDL, and HDL and could not explore the functions of the lipoproteins.

In conclusion, this study suggested positive causal effects of AS on TC, LDL, and HDL and negative causal effects of SLE on these cholesterol levels. No causal relationship was identified between RA and TC, LDL, and HDL. This study could be important for further exploration of the pathogenesis and management of rheumatic disease patients with a high risk of dyslipidemia, subsequently decreasing the long-term complications of these autoimmune diseases.

## Data availability statement

Publicly available datasets were analyzed in this study. This data can be found here: https://gwas.mrcieu.ac.uk/.

## Author contributions

GZ, YC and JL designed and performed the study. JZ, ZJ, LL, JS and RZ collected and analyzed the data. QS and XD interpreted the results. GZ wrote this paper. All authors contributed to the article and approved the submitted version.

## Funding

The present study was supported by the National Natural Science Foundation of China (grant nos. 82002311), Shaanxi Province Key Research and Development Project (grant nos. 2022 SF-192) and China Postdoctoral Science Foundation (grant nos. 2021M692575).

## Acknowledgments

We thank the GWAS summary datasets.

## Conflict of interest

The authors declare that the research was conducted in the absence of any commercial or financial relationships that could be construed as a potential conflict of interest.

## Publisher’s note

All claims expressed in this article are solely those of the authors and do not necessarily represent those of their affiliated organizations, or those of the publisher, the editors and the reviewers. Any product that may be evaluated in this article, or claim that may be made by its manufacturer, is not guaranteed or endorsed by the publisher.

## Abbreviations

AS, ankylosing spondylitis; RA, rheumatoid arthritis; SLE, systemic lupus erythematosus; MR, Mendelian randomization; TC, total cholesterol; LDL, low-density lipoprotein; HDL, high-density lipoprotein; IVs, instrumental variables; SNPs, single-nucleotide polymorphisms; GWAS, genome-wide association study; IVW, inverse variance weighted; β, beta.

## References

[1] Goldblatt F O'Neill SG . Clinical aspects of autoimmune rheumatic diseases. Lancet (2013) 382(9894):797–808. doi: 10.1016/S0140-6736(13)61499-3 23993190

[2] Sieper J Poddubnyy D . Axial spondyloarthritis. Lancet (2017) 390(10089):73–84. doi: 10.1016/s0140-6736(16)31591-4 28110981

[3] Costantino F Talpin A Said-Nahal R Goldberg M Henny J Chiocchia G . Prevalence of spondyloarthritis in reference to hla-B27 in the French population: Results of the gazel cohort. Ann Rheum Dis (2015) 74(4):689–93. doi: 10.1136/annrheumdis-2013-204436 24351517

[4] Bakland G Alsing R Singh K Nossent JC . Assessment of spondyloarthritis international society criteria for axial spondyloarthritis in chronic back pain patients with a high prevalence of hla-B27. Arthritis Care Res (Hoboken) (2013) 65(3):448–53. doi: 10.1002/acr.21804 22833469

[5] Smolen JS Aletaha D McInnes IB . Rheumatoid arthritis. Lancet (2016) 388(10055):2023–38. doi: 10.1016/S0140-6736(16)30173-8 27156434

[6] Kiriakidou M Ching CL . Systemic lupus erythematosus. Ann Intern Med (2020) 172(11):ITC81–96. doi: 10.7326/AITC202006020 32479157

[7] Durcan L O'Dwyer T Petri M . Management strategies and future directions for systemic lupus erythematosus in adults. Lancet (2019) 393(10188):2332–43. doi: 10.1016/S0140-6736(19)30237-5 31180030

[8] Peters MJ van Eijk IC Smulders YM Serne E Dijkmans BA van der Horst-Bruinsma IE . Signs of accelerated preclinical atherosclerosis in patients with ankylosing spondylitis. J Rheumatol (2010) 37(1):161–6. doi: 10.3899/jrheum.090667 19955053

[9] Avina-Zubieta JA Thomas J Sadatsafavi M Lehman AJ Lacaille D . Risk of incident cardiovascular events in patients with rheumatoid arthritis: A meta-analysis of observational studies. Ann Rheum Dis (2012) 71(9):1524–9. doi: 10.1136/annrheumdis-2011-200726 22425941

[10] Ference BA Graham I Tokgozoglu L Catapano AL . Impact of lipids on cardiovascular health: Jacc health promotion series. J Am Coll Cardiol (2018) 72(10):1141–56. doi: 10.1016/j.jacc.2018.06.046 30165986

[11] Semb AG Kvien TK DeMicco DA Fayyad R Wun CC LaRosa J . Prediction of cardiovascular events in patients with ankylosing spondylitis and psoriatic arthritis: Role of lipoproteins in a high-risk population. J Rheumatol (2012) 39(7):1433–40. doi: 10.3899/jrheum.111307 22660802

[12] Gkolfinopoulou C Stratikos E Theofilatos D Kardassis D Voulgari PV Drosos AA . Impaired antiatherogenic functions of high-density lipoprotein in patients with ankylosing spondylitis. J Rheumatol (2015) 42(9):1652–60. doi: 10.3899/jrheum.141532 26233507

[13] Kucuk A Ugur Uslu A Icli A Cure E Arslan S Turkmen K . The Ldl/Hdl ratio and atherosclerosis in ankylosing spondylitis. Z Rheumatol (2017) 76(1):58–63. doi: 10.1007/s00393-016-0092-4 27312464

[14] Boyer JF Gourraud PA Cantagrel A Davignon JL Constantin A . Traditional cardiovascular risk factors in rheumatoid arthritis: A meta-analysis. Joint Bone Spine (2011) 78(2):179–83. doi: 10.1016/j.jbspin.2010.07.016 20851020

[15] Liao KP Cai T Gainer VS Cagan A Murphy SN Liu C . Lipid and lipoprotein levels and trend in rheumatoid arthritis compared to the general population. Arthritis Care Res (Hoboken) (2013) 65(12):2046–50. doi: 10.1002/acr.22091 PMC4060244 23925980

[16] Atta AM Silva JPCG Santiago MB Oliveira IS Oliveira RC Sousa Atta MLB . Clinical and laboratory aspects of dyslipidemia in Brazilian women with systemic lupus erythematosus. Clin Rheumatol (2018) 37(6):1539–46. doi: 10.1007/s10067-018-4051-0 29516281

[17] Reiss AB Jacob B Ahmed S Carsons SE DeLeon J . Understanding accelerated atherosclerosis in systemic lupus erythematosus: Toward better treatment and prevention. Inflammation (2021) 44(5):1663–82. doi: 10.1007/s10753-021-01455-6 33821395

[18] Yarmolinsky J Wade KH Richmond RC Langdon RJ Bull CJ Tilling KM . Causal inference in cancer epidemiology: What is the role of mendelian randomization? Cancer Epidemiol Biomarkers Prev (2018) 27(9):995–1010. doi: 10.1158/1055-9965.EPI-17-1177 PMC6522350 29941659

[19] Burgess S Small DS Thompson SG . A review of instrumental variable estimators for mendelian randomization. Stat Methods Med Res (2017) 26(5):2333–55. doi: 10.1177/0962280215597579 PMC5642006 26282889

[20] Lawlor DA Harbord RM Sterne JAC Timpson N Davey Smith G . Mendelian randomization: Using genes as instruments for making causal inferences in epidemiology. Stat Med (2008) 27(8):1133–63. doi: 10.1002/sim.3034 17886233

[21] Cortes A Hadler J Pointon JP Robinson PC Karaderi T Leo P . Identification of multiple risk variants for ankylosing spondylitis through high-density genotyping of immune-related loci. Nat Genet (2013) 45(7):730–8. doi: 10.1038/ng.2667 PMC3757343 23749187

[22] Okada Y Wu D Trynka G Raj T Terao C Ikari K . Genetics of rheumatoid arthritis contributes to biology and drug discovery. Nature (2014) 506(7488):376–81. doi: 10.1038/nature12873 PMC3944098 24390342

[23] Bentham J Morris DL Graham DSC Pinder CL Tombleson P Behrens TW . Genetic association analyses implicate aberrant regulation of innate and adaptive immunity genes in the pathogenesis of systemic lupus erythematosus. Nat Genet (2015) 47(12):1457–64. doi: 10.1038/ng.3434 PMC4668589 26502338

[24] Willer CJ Schmidt EM Sengupta S Peloso GM Gustafsson S Kanoni S . Discovery and refinement of loci associated with lipid levels. Nat Genet (2013) 45(11):1274–83. doi: 10.1038/ng.2797 PMC3838666 24097068

[25] Hartwig FP Davies NM Hemani G Davey Smith G . Two-sample mendelian randomization: Avoiding the downsides of a powerful, widely applicable but potentially fallible technique. Int J Epidemiol (2016) 45(6):1717–26. doi: 10.1093/ije/dyx028 PMC5722032 28338968

[26] Bowden J Davey Smith G Haycock PC Burgess S . Consistent estimation in mendelian randomization with some invalid instruments using a weighted median estimator. Genet Epidemiol (2016) 40(4):304–14. doi: 10.1002/gepi.21965 PMC4849733 27061298

[27] Bowden J Davey Smith G Burgess S . Mendelian randomization with invalid instruments: Effect estimation and bias detection through egger regression. Int J Epidemiol (2015) 44(2):512–25. doi: 10.1093/ije/dyv080 PMC4469799 26050253

[28] Bowden J Del Greco MF Minelli C Davey Smith G Sheehan N Thompson J . A framework for the investigation of pleiotropy in two-sample summary data mendelian randomization. Stat Med (2017) 36(11):1783–802. doi: 10.1002/sim.7221 PMC5434863 28114746

[29] Zhao SS Robertson S Reich T Harrison NL Moots RJ Goodson NJ . Prevalence and impact of comorbidities in axial spondyloarthritis: Systematic review and meta-analysis. Rheumatology (2020) 59(Suppl4):iv47–57. doi: 10.1093/rheumatology/keaa246 PMC7566561 33053193

[30] García-Gómez C Martín-Martínez MA Fernández-Carballido C Castañeda S González-Juanatey C Sanchez-Alonso F . Hyperlipoproteinaemia(a) in patients with spondyloarthritis: Results of the cardiovascular in rheumatology (Carma) project. Clin Exp Rheumatol (2019) 37(5):774–82. 30789151

[31] Mathieu S Gossec L Dougados M Soubrier M . Cardiovascular profile in ankylosing spondylitis: A systematic review and meta-analysis. Arthritis Care Res (Hoboken) (2011) 63(4):557–63. doi: 10.1002/acr.20364 20890982

[32] van Halm VP van Denderen JC Peters MJL Twisk JWR van der Paardt M van der Horst-Bruinsma IE . Increased disease activity is associated with a deteriorated lipid profile in patients with ankylosing spondylitis. Ann Rheum Dis (2006) 65(11):1473–7. doi: 10.1136/ard.2005.050443 PMC1798360 16644785

[33] Olama SM Elarman MM . Evaluation of paraoxonase and arylesterase activities in Egyptian patients with ankylosing spondylitis. Rheumatol Int (2013) 33(6):1487–94. doi: 10.1007/s00296-012-2591-1 23239038

[34] Myasoedova E Crowson CS Kremers HM Fitz-Gibbon PD Therneau TM Gabriel SE . Total cholesterol and ldl levels decrease before rheumatoid arthritis. Ann Rheum Dis (2010) 69(7):1310–4. doi: 10.1136/ard.2009.122374 PMC2890039 19854708

[35] van Halm VP Nielen MMJ Nurmohamed MT van Schaardenburg D Reesink HW Voskuyl AE . Lipids and inflammation: Serial measurements of the lipid profile of blood donors who later developed rheumatoid arthritis. Ann Rheum Dis (2007) 66(2):184–8. doi: 10.1136/ard.2006.051672 PMC1798498 16760255

[36] van Boheemen L van Beers-Tas MH Kroese JM van de Stadt LA van Schaardenburg D Nurmohamed MT . Cardiovascular risk in persons at risk of developing rheumatoid arthritis. PloS One (2020) 15(8):e0237072. doi: 10.1371/journal.pone.0237072 32745151 PMC7398549

[37] Lauper K Gabay C . Cardiovascular risk in patients with rheumatoid arthritis. Semin Immunopathol (2017) 39(4):447–59. doi: 10.1007/s00281-017-0632-2 28455580

[38] Semb AG Ikdahl E Wibetoe G Crowson C Rollefstad S . Atherosclerotic cardiovascular disease prevention in rheumatoid arthritis. Nat Rev Rheumatol (2020) 16(7):361–79. doi: 10.1038/s41584-020-0428-y 32494054

[39] Bag-Ozbek A Giles JT . Inflammation, adiposity, and atherogenic dyslipidemia in rheumatoid arthritis: Is there a paradoxical relationship? Curr Allergy Asthma Rep (2015) 15(2):497. doi: 10.1007/s11882-014-0497-6 25504261

[40] Kostopoulou M Nikolopoulos D Parodis I Bertsias G . Cardiovascular disease in systemic lupus erythematosus: Recent data on epidemiology, risk factors and prevention. Curr Vasc Pharmacol (2020) 18(6):549–65. doi: 10.2174/1570161118666191227101636 31880245

[41] Fernández-Nebro A Marenco JL López-Longo F Galindo M Hernández-Cruz BE Narváez J . The effects of rituximab on the lipid profile of patients with active systemic lupus erythematosus: Results from a nationwide cohort in Spain (Lesimab). Lupus (2014) 23(10):1014–22. doi: 10.1177/0961203314534909 24833667

[42] Elfving P Puolakka K Kautiainen H Virta LJ Pohjolainen T Kaipiainen-Seppänen O . Drugs used in incident systemic lupus erythematosus - results from the Finnish nationwide register 2000-2007. Lupus (2016) 25(6):666–70. doi: 10.1177/0961203316628998 26821964

[43] Westerterp M Gautier EL Ganda A Molusky MM Wang W Fotakis P . Cholesterol accumulation in dendritic cells links the inflammasome to acquired immunity. Cell Metab (2017) 25(6):1294–304.e6. doi: 10.1016/j.cmet.2017.04.005 PMC5514787 28479366

[44] Halacoglu J Shea LA . Cardiovascular risk assessment and therapeutic implications in rheumatoid arthritis. J Cardiovasc Transl Res (2020) 13(5):878–90. doi: 10.1007/s12265-020-09964-9 32080804

[45] Navarini L Bisogno T Margiotta DPE Piccoli A Angeletti S Laudisio A . Role of the specialized proresolving mediator resolvin D1 in systemic lupus erythematosus: Preliminary results. J Immunol Res (2018) 2018:5264195. doi: 10.1155/2018/5264195 30420970 PMC6215556

