# Supplementary material for: Causal relationships between rheumatism and dyslipidemia: A two-sample Mendelian randomization study
Source: Front Endocrinol (Lausanne). 2022 Aug 31;13:961505. doi: 10.3389/fendo.2022.961505 (PMC9470850; doi:10.3389/fendo.2022.961505)
Supplement: Supplementary file 1 [file DataSheet_1.pdf]

Supplementary Figures

Figure S1 MR results of AS on TC.
Figure S2 MR results of AS on LDL.
Figure S3 MR results of AS on HDL.
Figure S4 MR results of RA on TC.
Figure S5 MR results of RA on LDL.
Figure S6 MR results of RA on HDL.
Figure S7 MR results of SLE on TC.
Figure S8 MR results of SLE on LDL.
Figure S9 MR results of SLE on HDL.
(MR: Mendelian Randomization; SNPs: single-nucleotide polymorphisms; AS: Ankylosing Spondylitis; RA: rheumatoid arthritis; SLE: systemic lupus erythematosus; TC: Total cholesterol; LDL: low density lipoprotein; HDL: high density lipoprotein)

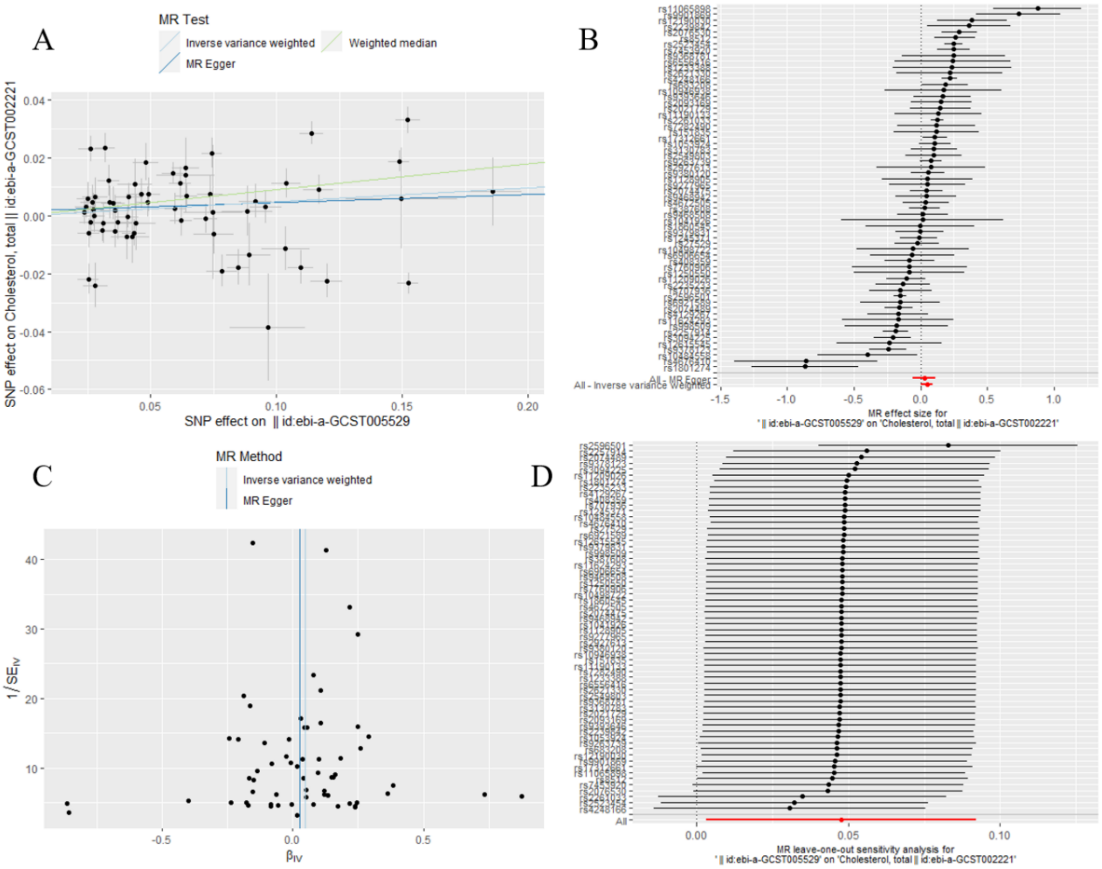

Figure S1 MR results of AS on TC. A: Scatter plot of SNPs associated with AS and TC; B: Forest plot of SNPs associated with AS and TC; C: Funnel plot of SNPs associated with AS and TC; D: Leave-one-out analysis of SNPs associated with AS and TC.

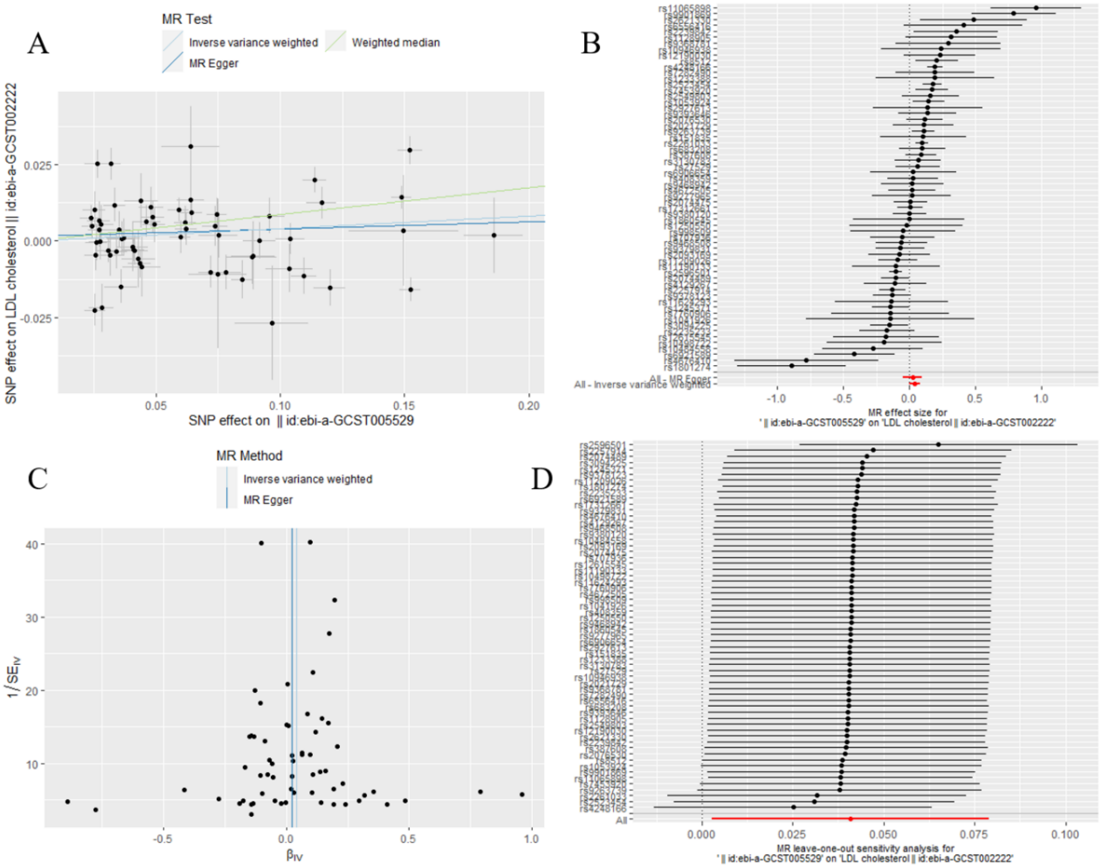

Figure S2 MR results of AS on LDL. A: Scatter plot of SNPs associated with AS and LDL; B: Forest plot of SNPs associated with AS and LDL; C: Funnel plot of SNPs associated with AS and LDL; D: Leave-one-out analysis of SNPs associated with AS and LDL.

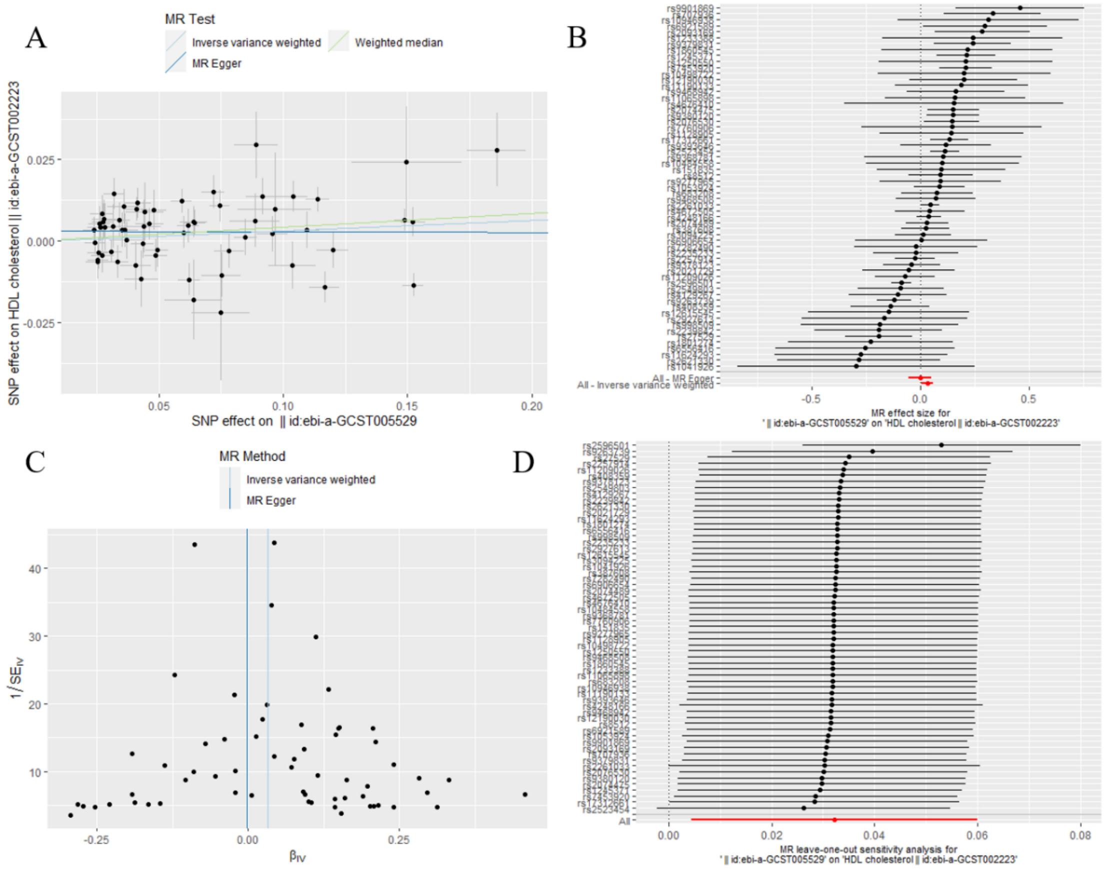

Figure S3 MR results of AS on HDL. A: Scatter plot of SNPs associated with AS and HDL; B: Forest plot of SNPs associated with AS and HDL; C: Funnel plot of SNPs associated with AS and HDL; D: Leave-one-out analysis of SNPs associated with AS and HDL.

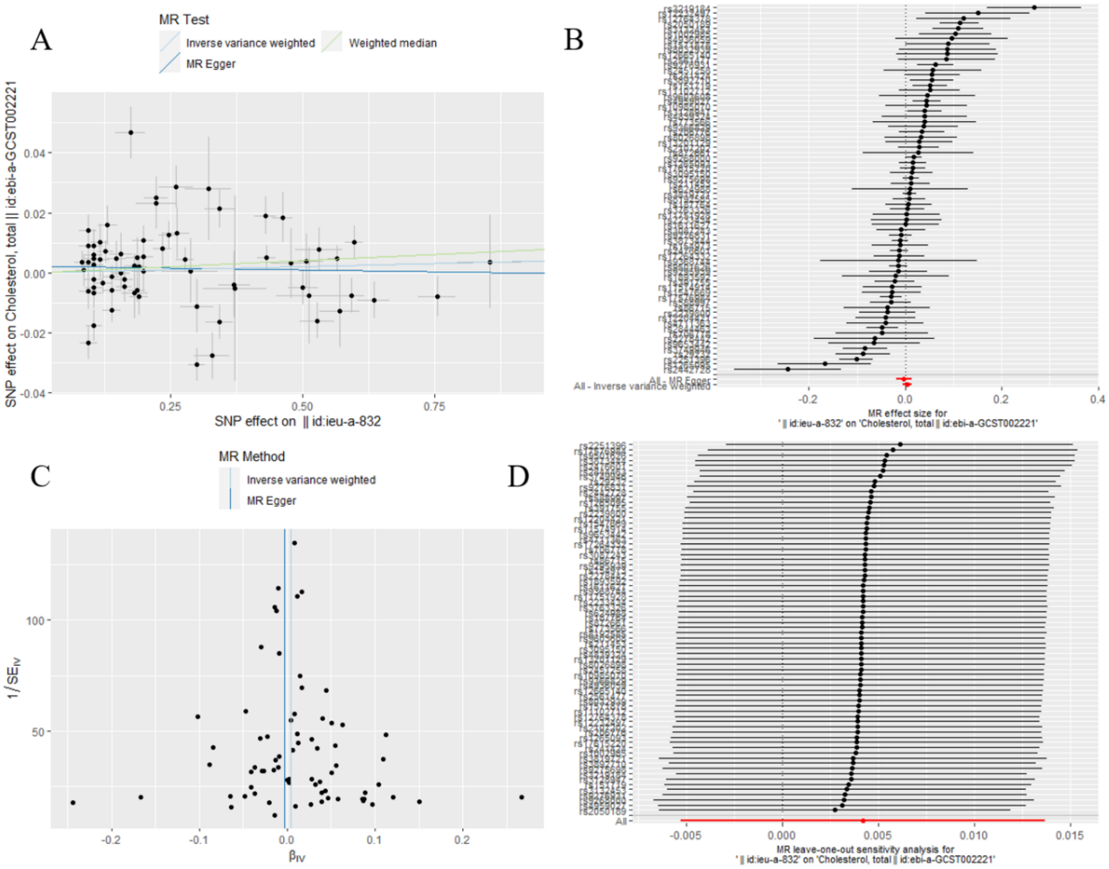

Figure S4 MR results of RA on TC. A: Scatter plot of SNPs associated with RA and TC; B: Forest plot of SNPs associated with RA and TC; C: Funnel plot of SNPs associated with RA and TC; D: Leave-one-out analysis of SNPs associated with RA and TC.

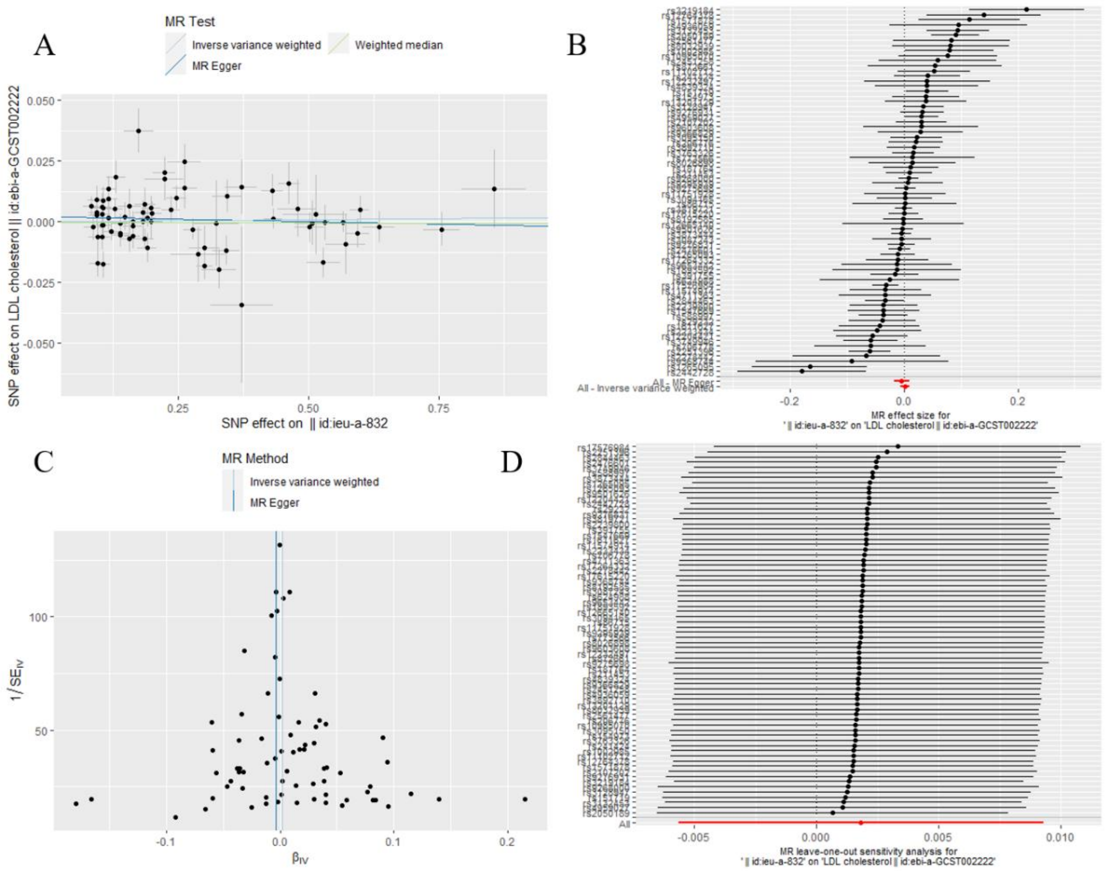

Figure S5 MR results of RA on LDL. A: Scatter plot of SNPs associated with RA and LDL; B: Forest plot of SNPs associated with RA and LDL; C: Funnel plot of SNPs associated with RA and LDL; D: Leave-one-out analysis of SNPs associated with RA and LDL.

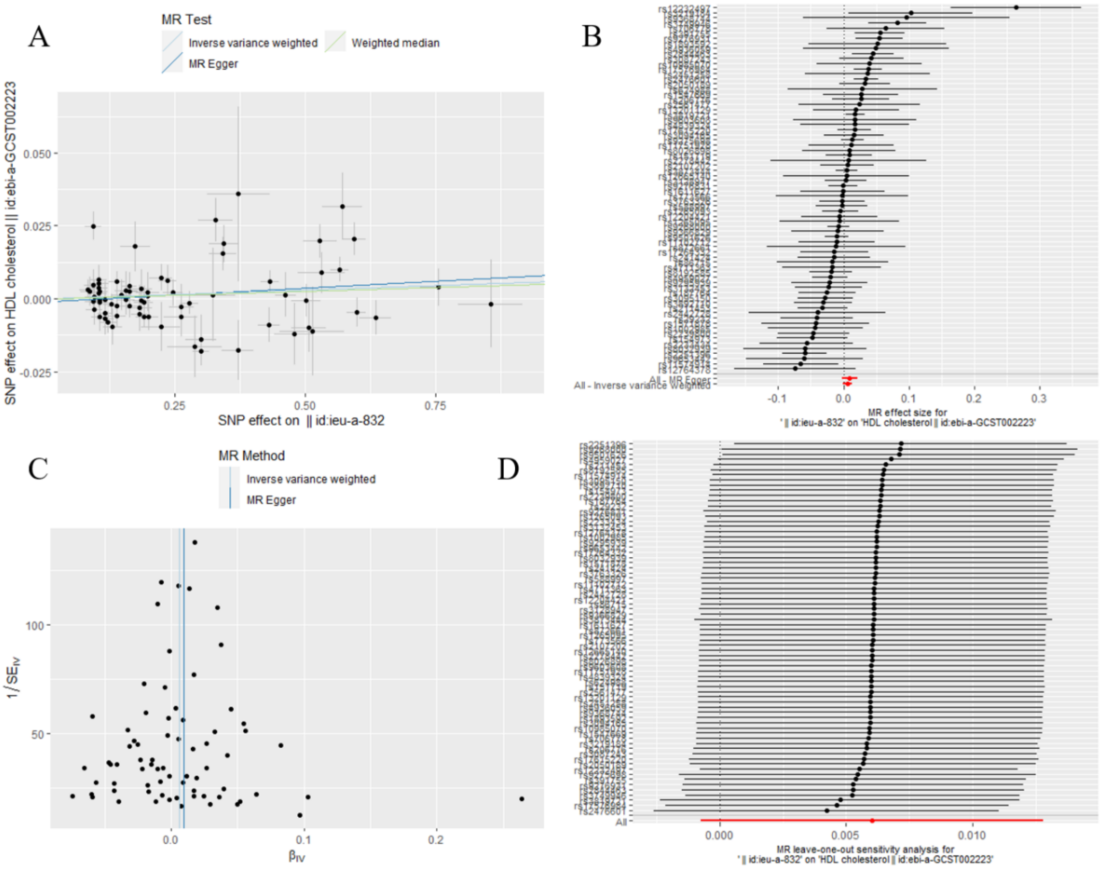

Figure S6 MR results of RA on HDL. A: Scatter plot of SNPs associated with RA and HDL; B: Forest plot of SNPs associated with RA and HDL; C: Funnel plot of SNPs associated with RA and HDL; D: Leave-one-out analysis of SNPs associated with RA and HDL.

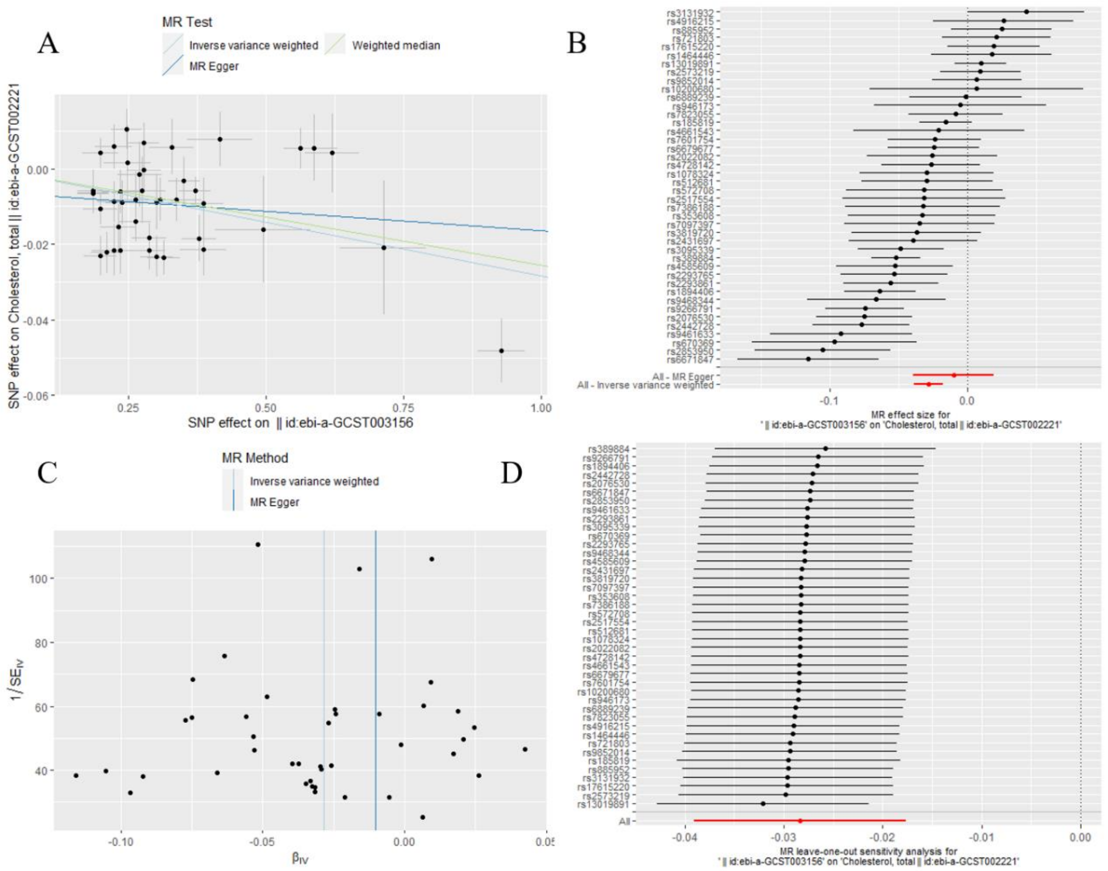

Figure S7 MR results of SLE on TC. A: Scatter plot of SNPs associated with SLE and TC; B: Forest plot of SNPs associated with SLE and TC; C: Funnel plot of SNPs associated with SLE and TC; D: Leave-one-out analysis of SNPs associated with SLE and TC.

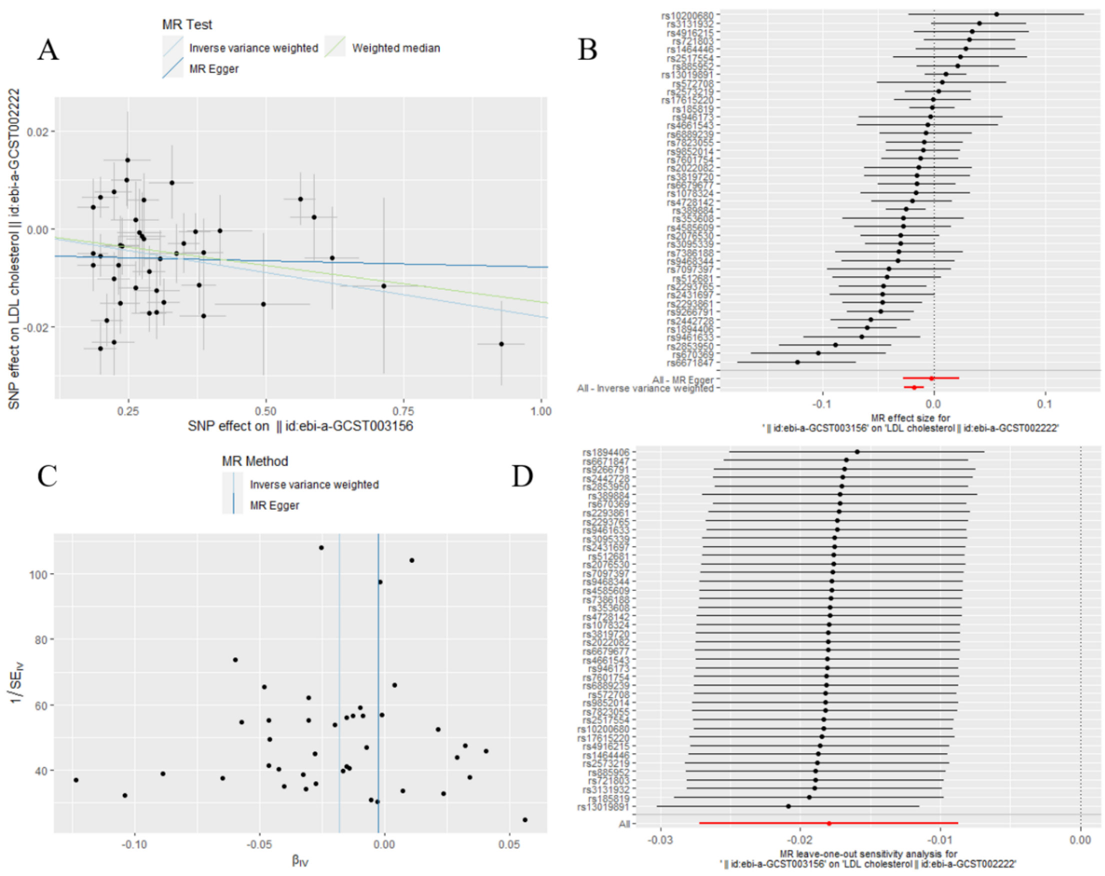

Figure S8 MR results of SLE on LDL. A: Scatter plot of SNPs associated with SLE and LDL; B: Forest plot of SNPs associated with SLE and LDL; C: Funnel plot of SNPs associated with SLE and LDL; D: Leave-one-out analysis of SNPs associated with SLE and LDL.

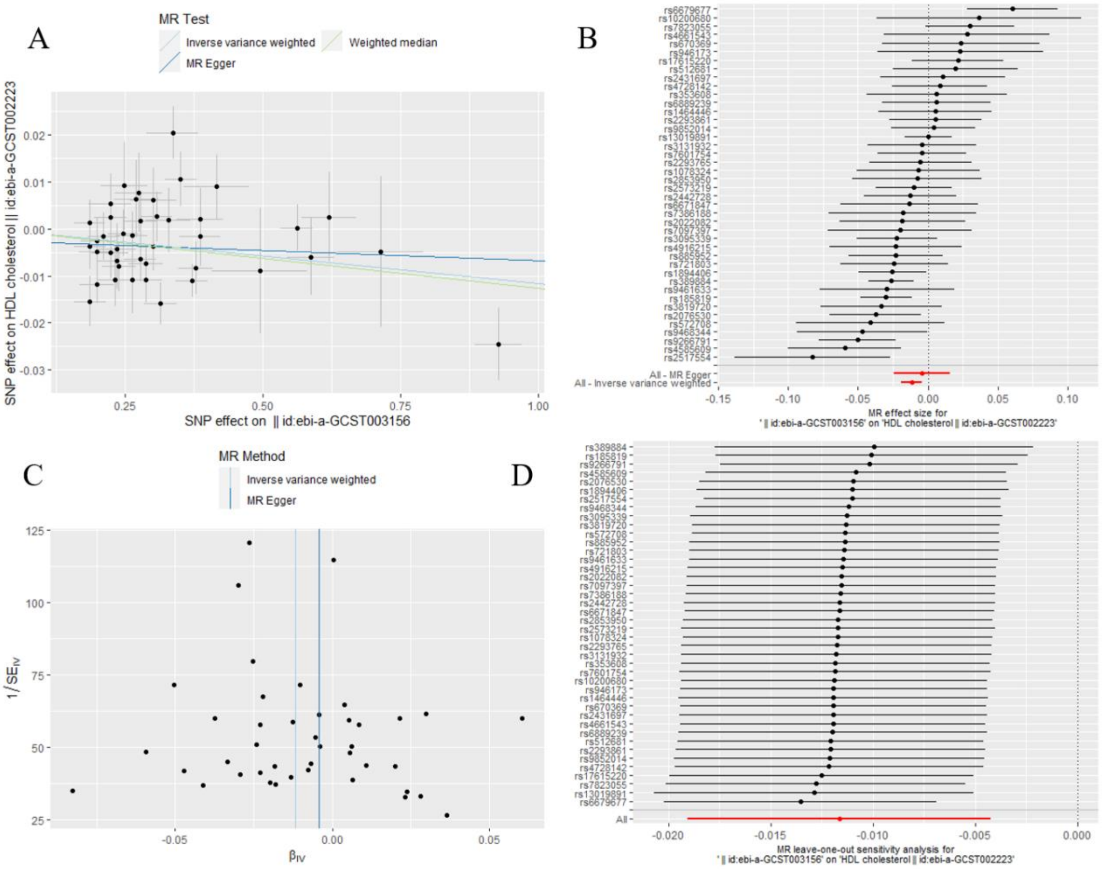

Figure S9 MR results of SLE on HDL. A: Scatter plot of SNPs associated with SLE and HDL; B: Forest plot of SNPs associated with SLE and HDL; C: Funnel plot of SNPs associated with SLE and HDL; D: Leave-one-out analysis of SNPs associated with SLE and HDL.
