# Supplementary material for: Causal relationships between rheumatism and dyslipidemia: A two-sample Mendelian randomization study
Source: Front Endocrinol (Lausanne). 2022 Aug 31;13:961505. doi: 10.3389/fendo.2022.961505 (PMC9470850; doi:10.3389/fendo.2022.961505)
Supplement: Supplementary file 2 [file DataSheet_2.pdf]

Table S1 The information of SNPs selected for MR analysis between AS and TC

Number	SNPs	*P*-value	Chromosome	Beta	SE	Sample size	EA	OA	*F*-value
1	rs1041926	1.55E-10	6	-0.0012	0.0232	94595	A	G	40.96173
2	rs10484558	7.93E-11	6	0.0386	0.0184	94595	C	T	42.2758
3	rs10498722	1.24E-08	6	0.0025	0.0095	94595	T	C	32.42481
4	rs1053924	7.09E-50	6	-0.0068	0.0039	94595	C	T	220.4847
5	rs10946938	8.81E-09	6	-0.0046	0.006	94595	A	C	33.08798
6	rs11065898	4.71E-08	12	0.023	0.0044	94595	T	C	29.83296
7	rs11190133	4.84E-14	10	-0.0045	0.0056	94595	T	C	56.7921
8	rs11209026	1.94E-27	1	0.0113	0.0076	94595	A	G	117.7744
9	rs1128905	6.95E-09	9	-0.0012	0.0041	94595	C	T	33.54906
10	rs11624293	1.49E-10	14	-0.0073	0.0092	94595	C	T	41.03961
11	rs12190030	1.22E-21	6	0.0184	0.0064	94595	T	C	91.32994
12	rs1233388	1.49E-08	6	0.0066	0.0063	94595	G	A	32.07078
13	rs1245371	5.06E-55	6	0.001	0.0051	94595	G	A	244.0845
14	rs1250550	1.46E-09	10	0.0022	0.0055	94595	A	C	36.58245
15	rs12615545	1.03E-09	2	-0.006	0.0051	94595	C	T	37.266
16	rs151835	9.69E-15	5	-0.0042	0.0057	94595	G	A	59.95739
17	rs17312661	1.56E-73	6	-0.0112	0.0049	94595	G	A	329.0356
18	rs1801274	1.35E-09	1	-0.0219	0.0052	94595	G	A	36.74092
19	rs1860545	2.78E-10	12	1.00E-04	0.0057	94595	A	G	39.82088
20	rs2021729	4.45E-26	6	0.0073	0.0057	94595	A	G	111.5632
21	rs2074475	1.09E-56	6	0.0082	0.0117	94595	C	T	251.7352
22	rs2074489	1.01E-103	6	0.0179	0.0058	94595	T	C	467.7104
23	rs2076530	1.53E-75	6	0.0216	0.0051	94595	C	T	338.2522
24	rs2093169	8.32E-14	6	-0.0064	0.0048	94595	T	C	55.72894
25	rs2235233	9.39E-21	6	-0.0059	0.0045	94595	C	T	87.28543
26	rs2239842	1.36E-14	6	0.0121	0.0053	94595	G	A	59.29114
27	rs2257914	4.32E-79	6	0.0225	0.0059	94595	A	C	354.5499
28	rs2261033	1.00E-200	6	0.0187	0.0036	94595	G	A	1309.706
29	rs2523454	1.54E-134	6	-0.0284	0.0039	94595	A	G	609.3619
31	rs2549803	2.79E-27	5	-0.0047	0.0052	94595	T	C	117.0588
32	rs2596501	1.00E-200	6	0.0233	0.0036	94595	T	C	1319.308
33	rs2621330	2.95E-08	6	-0.0139	0.013	94595	T	C	30.74041
34	rs27529	3.28E-47	5	0.0016	0.0053	94595	G	A	208.2702
35	rs2927613	7.07E-10	5	-0.0021	0.0057	94595	A	G	38.00118
36	rs3094225	1.12E-81	6	0.0178	0.006	94595	A	G	366.4394
37	rs3130783	5.45E-47	6	0.0074	0.0065	94595	A	G	207.2555
38	rs387608	1.59E-51	6	-0.0029	0.0056	94595	A	G	228.0515
39	rs408359	7.71E-22	6	0.0062	0.0071	94595	A	G	92.23078
40	rs4129267	3.32E-13	1	0.0052	0.0036	94595	T	C	53.00824
41	rs4248166	1.00E-200	6	0.033	0.0046	94595	C	T	956.0403
42	rs4672505	5.14E-47	2	-0.0023	0.0053	94595	G	A	207.3744
43	rs4676410	9.90E-09	2	-0.0241	0.0077	94595	A	G	32.86115
44	rs6556416	4.22E-08	5	0.006	0.0056	94595	C	A	30.04494
45	rs683208	7.58E-47	6	0.0113	0.0054	94595	C	T	206.6011
46	rs6906654	4.82E-08	6	0.0023	0.0059	94595	A	G	29.78965
47	rs6921589	2.42E-08	6	-0.0055	0.0054	94595	C	A	31.12054
48	rs707936	2.47E-24	6	0.0134	0.0107	94595	A	G	103.6029
49	rs7282490	6.21E-09	21	-0.0029	0.0036	94595	A	G	33.76682
50	rs7453920	1.18E-42	6	0.0146	0.0037	94595	G	A	187.384
51	rs7760906	1.66E-08	6	0.0026	0.0069	94595	A	C	31.86106
52	rs8512	6.55E-32	6	0.0165	0.005	94595	A	G	138.2128
53	rs9263739	7.25E-85	6	-0.009	0.005	94595	T	C	381.0747
54	rs9277965	3.39E-10	6	-0.0018	0.0053	94595	A	G	39.4373
55	rs9368781	9.02E-11	6	-0.0108	0.0087	94595	G	A	42.02379
56	rs9378123	9.75E-37	6	0.0191	0.0055	94595	G	A	160.2948
57	rs9379831	3.40E-20	6	-3.00E-04	0.0038	94595	A	C	84.74387
58	rs9380120	5.00E-46	6	0.005	0.0058	94595	C	T	202.8458
59	rs9393646	1.10E-16	6	0.0075	0.0051	94595	A	G	68.78744
60	rs9468508	1.88E-18	6	-0.0015	0.0086	94595	T	C	76.80947
61	rs9468942	1.08E-11	6	-0.006	0.0175	94595	A	G	46.17482
62	rs9901869	6.04E-15	17	0.0234	0.0051	94595	A	G	60.88986
63	rs998509	4.30E-11	5	0.0072	0.008	94595	A	G	43.47316

Table S2 The information of SNPs selected for MR analysis between AS and LDL

Number	SNPs	*P*-value	Chromosome	Beta	SE	Sample size	EA	OA	*F*-value
1	rs1041926	1.55E-10	6	0.0108	0.0244	94595	A	G	40.96173
2	rs10484558	7.93E-11	6	0.0268	0.0187	94595	C	T	42.2758
3	rs10498722	1.24E-08	6	0.0084	0.0098	94595	T	C	32.42481
4	rs1053924	7.09E-50	6	-0.0094	0.004	94595	C	T	220.4847
5	rs10946938	8.81E-09	6	-0.0065	0.0062	94595	A	C	33.08798
6	rs11065898	4.71E-08	12	0.0252	0.0046	94595	T	C	29.83296
7	rs11190133	4.84E-14	10	0.0034	0.0058	94595	T	C	56.7921
8	rs11209026	1.94E-27	1	0.009	0.0079	94595	A	G	117.7744
9	rs1128905	6.95E-09	9	-0.0075	0.0042	94595	C	T	33.54906
10	rs11624293	1.49E-10	14	-0.0058	0.0094	94595	C	T	41.03961
11	rs12190030	1.22E-21	6	0.0111	0.0066	94595	T	C	91.32994
12	rs1233388	1.49E-08	6	0.0054	0.0064	94595	G	A	32.07078
13	rs1245371	5.06E-55	6	0.0102	0.0052	94595	G	A	244.0845
14	rs1250550	1.46E-09	10	6.00E-04	0.0057	94595	A	C	36.58245
15	rs12615545	1.03E-09	2	-0.0045	0.0052	94595	C	T	37.266
16	rs151835	9.69E-15	5	-0.0037	0.0058	94595	G	A	59.95739
17	rs17312661	1.56E-73	6	-6.00E-04	0.005	94595	G	A	329.0356
18	rs1801274	1.35E-09	1	-0.0226	0.0053	94595	G	A	36.74092
19	rs1860545	2.78E-10	12	1.00E-04	0.0059	94595	A	G	39.82088
20	rs2021729	4.45E-26	6	0.0053	0.0058	94595	A	G	111.5632
21	rs2074475	1.09E-56	6	0.0018	0.0123	94595	C	T	251.7352
22	rs2074489	1.01E-103	6	0.0115	0.006	94595	T	C	467.7104
23	rs2076530	1.53E-75	6	0.0087	0.0052	94595	C	T	338.2522
24	rs2093169	8.32E-14	6	0.0031	0.0049	94595	T	C	55.72894
25	rs2235233	9.39E-21	6	-0.0074	0.0046	94595	C	T	87.28543
26	rs2239842	1.36E-14	6	0.0118	0.0054	94595	G	A	59.29114
27	rs2257914	4.32E-79	6	0.0153	0.006	94595	A	C	354.5499
28	rs2261033	1.00E-200	6	0.0143	0.0037	94595	G	A	1309.706
29	rs2523454	1.54E-134	6	-0.02	0.0041	94595	A	G	609.3619
31	rs2549803	2.79E-27	5	-0.0078	0.0054	94595	T	C	117.0588
32	rs2596501	1.00E-200	6	0.0159	0.0038	94595	T	C	1319.308
33	rs2621330	2.95E-08	6	-0.0309	0.0132	94595	T	C	30.74041
34	rs27529	3.28E-47	5	-0.004	0.0054	94595	G	A	208.2702
35	rs2927613	7.07E-10	5	-0.0038	0.0058	94595	A	G	38.00118
36	rs3094225	1.12E-81	6	0.0127	0.0062	94595	A	G	366.4394
37	rs3130783	5.45E-47	6	0.0048	0.0066	94595	A	G	207.2555
38	rs387608	1.59E-51	6	-0.0082	0.0057	94595	A	G	228.0515
39	rs408359	7.71E-22	6	-0.0019	0.0073	94595	A	G	92.23078
40	rs4129267	3.32E-13	1	0.0033	0.0037	94595	T	C	53.00824
41	rs4248166	1.00E-200	6	0.0296	0.0047	94595	C	T	956.0403
42	rs4672505	5.14E-47	2	-0.0013	0.0054	94595	G	A	207.3744
43	rs4676410	9.90E-09	2	-0.0219	0.0078	94595	A	G	32.86115
44	rs6556416	4.22E-08	5	0.0103	0.0058	94595	C	A	30.04494
45	rs683208	7.58E-47	6	0.0059	0.0055	94595	C	T	206.6011
46	rs6906654	4.82E-08	6	-0.0011	0.0061	94595	A	G	29.78965
47	rs6921589	2.42E-08	6	-0.0149	0.0056	94595	C	A	31.12054
48	rs707936	2.47E-24	6	0.0048	0.011	94595	A	G	103.6029
49	rs7282490	6.21E-09	21	-0.0047	0.0037	94595	A	G	33.76682
50	rs7453920	1.18E-42	6	0.0101	0.0038	94595	G	A	187.384
51	rs7760906	1.66E-08	6	0.0045	0.0071	94595	A	C	31.86106
52	rs8512	6.55E-32	6	0.0133	0.0052	94595	A	G	138.2128
53	rs9263739	7.25E-85	6	-0.0125	0.0052	94595	T	C	381.0747
54	rs9277965	3.39E-10	6	-7.00E-04	0.0055	94595	A	G	39.4373
55	rs9368781	9.02E-11	6	-0.013	0.0089	94595	G	A	42.02379
56	rs9378123	9.75E-37	6	0.0102	0.0057	94595	G	A	160.2948
57	rs9379831	3.40E-20	6	-0.0028	0.0039	94595	A	C	84.74387
58	rs9380120	5.00E-46	6	1.00E-04	0.006	94595	C	T	202.8458
59	rs9393646	1.10E-16	6	0.0064	0.0052	94595	A	G	68.78744
60	rs9468508	1.88E-18	6	0.0053	0.0089	94595	T	C	76.80947
61	rs9468942	1.08E-11	6	-0.0034	0.0181	94595	A	G	46.17482
62	rs9901869	6.04E-15	17	0.0252	0.0052	94595	A	G	60.88986
63	rs998509	4.30E-11	5	0.0019	0.0082	94595	A	G	43.47316

Table S3 The information of SNPs selected for MR analysis between AS and HDL

Number	SNPs	*P*-value	Chromosome	Beta	SE	Sample size	EA	OA	*F*-value
1	rs1041926	1.55E-10	6	0.022	0.0208	94595	A	G	40.96173
2	rs10484558	7.93E-11	6	-0.0097	0.0173	94595	C	T	42.2758
3	rs10498722	1.24E-08	6	-0.0089	0.0089	94595	T	C	32.42481
4	rs1053924	7.09E-50	6	-0.0057	0.0038	94595	C	T	220.4847
5	rs10946938	8.81E-09	6	-0.0084	0.0057	94595	A	C	33.08798
6	rs11065898	4.71E-08	12	0.0042	0.0043	94595	T	C	29.83296
7	rs11190133	4.84E-14	10	-0.0064	0.0053	94595	T	C	56.7921
8	rs11209026	1.94E-27	1	0.0074	0.0073	94595	A	G	117.7744
9	rs1128905	6.95E-09	9	-0.0034	0.004	94595	C	T	33.54906
10	rs11624293	1.49E-10	14	-0.0117	0.0087	94595	C	T	41.03961
11	rs12190030	1.22E-21	6	0.0095	0.0061	94595	T	C	91.32994
12	rs1233388	1.49E-08	6	0.0067	0.0059	94595	G	A	32.07078
13	rs1245371	5.06E-55	6	-0.0151	0.005	94595	G	A	244.0845
14	rs1250550	1.46E-09	10	-0.0054	0.0053	94595	A	C	36.58245
15	rs12615545	1.03E-09	2	-0.0037	0.0048	94595	C	T	37.266
16	rs151835	9.69E-15	5	-0.0033	0.0053	94595	G	A	59.95739
17	rs17312661	1.56E-73	6	-0.0138	0.0047	94595	G	A	329.0356
18	rs1801274	1.35E-09	1	-0.0058	0.0049	94595	G	A	36.74092
19	rs1860545	2.78E-10	12	-0.0059	0.0055	94595	A	G	39.82088
20	rs2021729	4.45E-26	6	-0.0027	0.0053	94595	A	G	111.5632
21	rs2074475	1.09E-56	6	0.028	0.0112	94595	C	T	251.7352
22	rs2074489	1.01E-103	6	-0.0034	0.0055	94595	T	C	467.7104
23	rs2076530	1.53E-75	6	0.0108	0.0048	94595	C	T	338.2522
24	rs2093169	8.32E-14	6	-0.0117	0.0046	94595	T	C	55.72894
25	rs2235233	9.39E-21	6	-9.00E-04	0.0043	94595	C	T	87.28543
26	rs2239842	1.36E-14	6	-0.0064	0.005	94595	G	A	59.29114
27	rs2257914	4.32E-79	6	0.0028	0.0056	94595	A	C	354.5499
28	rs2261033	1.00E-200	6	0.0065	0.0034	94595	G	A	1309.706
29	rs2523454	1.54E-134	6	-0.0128	0.0038	94595	A	G	609.3619
31	rs2549803	2.79E-27	5	0.0044	0.0049	94595	T	C	117.0588
32	rs2596501	1.00E-200	6	0.0135	0.0035	94595	T	C	1319.308
33	rs2621330	2.95E-08	6	0.018	0.0122	94595	T	C	30.74041
34	rs27529	3.28E-47	5	0.0119	0.0049	94595	G	A	208.2702
35	rs2927613	7.07E-10	5	0.0045	0.0053	94595	A	G	38.00118
36	rs3094225	1.12E-81	6	-0.0011	0.0056	94595	A	G	366.4394
37	rs387608	1.59E-51	6	-0.0022	0.0054	94595	A	G	228.0515
38	rs408359	7.71E-22	6	0.0104	0.0069	94595	A	G	92.23078
39	rs4129267	3.32E-13	1	0.0032	0.0035	94595	T	C	53.00824
40	rs4248166	1.00E-200	6	0.0059	0.0044	94595	C	T	956.0403
41	rs4672505	5.14E-47	2	-0.0026	0.0049	94595	G	A	207.3744
42	rs4676410	9.90E-09	2	0.0043	0.0072	94595	A	G	32.86115
43	rs6556416	4.22E-08	5	-0.0064	0.0053	94595	C	A	30.04494
44	rs683208	7.58E-47	6	0.0047	0.0052	94595	C	T	206.6011
45	rs6906654	4.82E-08	6	-2.00E-04	0.0057	94595	A	G	29.78965
46	rs6921589	2.42E-08	6	0.0106	0.0052	94595	C	A	31.12054
47	rs707936	2.47E-24	6	-0.0295	0.0101	94595	A	G	103.6029
48	rs7282490	6.21E-09	21	5.00E-04	0.0035	94595	A	G	33.76682
49	rs7453920	1.18E-42	6	0.0122	0.0036	94595	G	A	187.384
50	rs7760906	1.66E-08	6	-0.0045	0.0066	94595	A	C	31.86106
51	rs8512	6.55E-32	6	0.0059	0.0048	94595	A	G	138.2128
52	rs9263739	7.25E-85	6	0.0141	0.0048	94595	T	C	381.0747
53	rs9277965	3.39E-10	6	-0.0033	0.0051	94595	A	G	39.4373
54	rs9368781	9.02E-11	6	-0.0046	0.0081	94595	G	A	42.02379
55	rs9378123	9.75E-37	6	0.0031	0.0053	94595	G	A	160.2948
56	rs9379831	3.40E-20	6	0.0098	0.0037	94595	A	C	84.74387
57	rs9380120	5.00E-46	6	0.0136	0.0056	94595	C	T	202.8458
58	rs9393646	1.10E-16	6	0.0053	0.0049	94595	A	G	68.78744
59	rs9468508	1.88E-18	6	-0.0063	0.0083	94595	T	C	76.80947
60	rs9468942	1.08E-11	6	-0.0243	0.0171	94595	A	G	46.17482
61	rs9901869	6.04E-15	17	0.0146	0.0048	94595	A	G	60.88986
62	rs998509	4.30E-11	5	0.0076	0.0075	94595	A	G	43.47316

Table S4 The information of SNPs selected for MR analysis between RA and TC

Number	SNPs	*P*-value	Chromosome	Beta	SE	Sample size	EA	OA	*F*-value
1	rs1002985	3.40E-09	6	-0.0232	0.0086	94595	T	C	34.94035492
2	rs10985070	1.70E-08	9	-0.0036	0.0036	94595	A	C	31.81033859
3	rs11102712	1.00E-28	1	0.0133	0.0084	94595	T	C	123.6594827
4	rs11574914	1.50E-13	9	-0.0033	0.0038	94595	A	G	54.57048351
5	rs11751928	7.60E-19	6	-5.00E-04	0.007	94595	T	C	78.60156425
6	rs12204421	1.60E-26	6	0.0079	0.006	94595	G	A	113.5924139
7	rs12232497	3.60E-09	17	0.0142	0.0052	94595	C	T	34.82901639
8	rs1265093	1.20E-62	6	0.0045	0.004	94595	A	G	279.0669218
9	rs1265095	8.70E-13	6	0.0176	0.0052	94595	A	G	51.11767248
10	rs12665140	1.60E-13	6	0.028	0.0172	94595	A	C	54.44348633
11	rs12764378	1.90E-13	10	0.0158	0.0065	94595	A	G	54.10525646
12	rs13201129	1.50E-20	6	0.0052	0.0066	94595	C	T	86.36050112
13	rs13330176	9.00E-09	16	0.004	0.008	94595	A	T	33.04637273
14	rs151719	4.80E-38	6	0.0126	0.0046	94595	T	C	166.2819667
15	rs1547669	1.60E-27	6	-0.0046	0.0051	94595	G	A	118.1592702
16	rs154973	2.70E-34	6	0.0039	0.011	94595	T	C	149.1201337
17	rs154977	5.60E-64	6	0.0106	0.0047	94595	G	C	285.1741339
18	rs1571878	4.90E-15	6	-0.0103	0.0052	94595	T	C	61.30077778
19	rs1611627	1.30E-21	6	-1.00E-04	0.0056	94595	C	T	91.19816669
20	rs17264332	7.10E-19	6	-0.0021	0.0044	94595	G	A	78.73552445
21	rs17576984	2.80E-60	6	0.0159	0.006	94595	T	C	268.1999285
22	rs17615220	1.40E-32	6	-0.0078	0.0071	94595	G	A	141.2764235
23	rs1858037	5.90E-10	2	0.0073	0.0053	94595	A	T	38.35468196
24	rs187764	4.00E-36	6	-0.0032	0.0116	94595	T	C	157.4902441
25	rs1893592	9.80E-09	21	0.0021	0.0059	94595	C	A	32.8805961
26	rs2050189	7.40E-25	6	-0.0251	0.0046	94595	C	T	105.9937523
27	rs206776	3.30E-42	6	0.008	0.0056	94595	C	T	185.3447654
28	rs2107202	3.40E-26	6	0.0055	0.0043	94595	C	T	112.0986979
29	rs211453	2.20E-32	6	-0.0021	0.0039	94595	G	T	140.3784433
30	rs2233434	3.30E-08	6	7.00E-04	0.0108	94595	G	A	30.52262393
31	rs2239800	2.50E-23	6	0.0112	0.0089	94595	G	A	99.02027042
32	rs2244579	1.10E-28	6	0.0224	0.0059	94595	C	G	123.4708391
33	rs2251396	1.60E-77	6	-0.0305	0.0053	94595	A	G	347.3526648
34	rs2278442	2.30E-08	19	-0.0061	0.0061	94595	A	G	31.22328226
35	rs241424	1.60E-16	6	-0.0071	0.0037	94595	A	G	68.0423785
36	rs2442728	2.60E-09	6	-0.0232	0.0054	94595	T	G	35.46278441
37	rs2451258	6.60E-10	6	0.006	0.0054	94595	T	C	38.13561104
38	rs2476601	1.60E-149	1	0.0075	0.0057	94595	G	A	678.2551357
39	rs2561477	5.20E-10	5	-0.009	0.0054	94595	A	G	38.60115167
40	rs2844463	9.50E-40	6	0.0163	0.0058	94595	A	G	174.0817865
41	rs29232	1.20E-16	6	0.0123	0.004	94595	T	C	68.60964345
42	rs3087243	9.20E-20	2	0.0013	0.0036	94595	A	G	82.77339454
43	rs3095150	5.70E-29	6	0.0023	0.0041	94595	T	C	124.7760995
44	rs3128947	2.00E-115	6	0.0185	0.0083	94595	G	A	521.495605
45	rs3132453	1.10E-15	6	0.0287	0.007	94595	G	T	64.24291302
46	rs3219184	6.40E-10	6	0.0466	0.0087	94595	G	A	38.19526887
47	rs3749946	4.00E-23	6	0.0276	0.0077	94595	A	C	98.08874685
48	rs3763326	2.40E-44	6	-0.0037	0.0156	94595	C	T	195.1411272
49	rs3819721	1.00E-200	6	0.0049	0.0042	94595	A	G	1106.619901
50	rs3873444	6.10E-103	6	0.0078	0.0066	94595	T	C	464.1208275
51	rs3892710	1.90E-23	6	0.0109	0.0046	94595	T	C	99.56291875
52	rs391755	1.50E-50	6	0.0128	0.012	94595	G	A	223.5779176
53	rs4452313	2.70E-10	3	-0.009	0.0056	94595	T	A	39.88086888
54	rs4673266	4.50E-11	2	0.0089	0.006	94595	T	A	43.38373104
55	rs4711363	8.90E-15	6	0.0058	0.0057	94595	G	A	60.12520638
56	rs4839324	6.50E-13	1	0.0062	0.0071	94595	A	G	51.69013224
57	rs4936059	4.60E-08	11	0.0091	0.0056	94595	G	A	29.87840789
58	rs4959027	6.60E-95	6	0.0191	0.0063	94595	G	A	427.2028705
59	rs588997	2.20E-30	6	0.0057	0.004	94595	A	G	131.2357303
60	rs624988	4.60E-08	1	-8.00E-04	0.0053	94595	C	T	29.87842993
61	rs706778	7.10E-12	10	-0.005	0.0051	94595	T	C	46.99981791
62	rs773566	4.80E-12	1	0.0046	0.0064	94595	C	T	47.76704196
63	rs8026898	2.40E-17	15	0.0048	0.0057	94595	A	G	71.78450094
64	rs8032939	2.40E-12	15	0.0102	0.006	94595	C	T	49.12630299
65	rs8192585	3.50E-52	6	0.004	0.0088	94595	A	G	231.0606429
66	rs86715	1.10E-13	6	0.0068	0.0083	94595	G	A	55.17983407
67	rs872661	1.30E-08	1	0.0028	0.0062	94595	G	A	32.3316359
68	rs915654	1.60E-79	6	0.0123	0.0039	94595	A	T	356.5347826
69	rs9268000	1.00E-200	6	0.0102	0.0053	94595	C	A	1143.795691
70	rs9275698	1.50E-139	6	-0.005	0.0039	94595	G	A	632.4024383
71	rs9276831	8.40E-72	6	0.0048	0.0059	94595	G	A	321.0858045
72	rs9276931	3.10E-33	6	-0.0215	0.0065	94595	G	A	144.2709554
73	rs9295939	1.60E-16	6	0.0076	0.0159	94595	G	A	68.04276205
74	rs9366829	1.30E-09	6	0.0035	0.0035	94595	G	A	36.81353628
75	rs9368744	5.80E-10	6	-0.0052	0.0307	94595	T	C	38.38768475
76	rs9501626	3.10E-97	6	0.009	0.006	94595	A	C	437.9002258
77	rs9603608	7.60E-11	13	-0.0048	0.0053	94595	C	A	42.35801786
78	rs9653442	3.60E-12	2	0.0068	0.0051	94595	T	C	48.33154658

Table S5 The information of SNPs selected for MR analysis between RA and LDL

Number	SNPs	P-value	Chromosome	Beta	SE	Sample size	EA	OA	F-value
1	rs1002985	3.40E-09	6	-0.0177	0.0089	94595	T	C	34.94035
2	rs10985070	1.70E-08	9	-0.0064	0.0037	94595	A	C	31.81034
3	rs11102712	1.00E-28	1	0.0139	0.0085	94595	T	C	123.6595
4	rs11574914	1.50E-13	9	-0.004	0.0039	94595	A	G	54.57048
5	rs11751928	7.60E-19	6	-3.00E-04	0.0072	94595	T	C	78.60156
6	rs12204421	1.60E-26	6	0.0107	0.0061	94595	G	A	113.5924
7	rs12232497	3.60E-09	17	0.0038	0.0053	94595	C	T	34.82902
8	rs1265093	1.20E-62	6	-0.0031	0.0042	94595	A	G	279.0669
9	rs1265095	8.70E-13	6	0.0175	0.0054	94595	A	G	51.11767
10	rs12665140	1.60E-13	6	-6.00E-04	0.0176	94595	A	C	54.44349
11	rs12764378	1.90E-13	10	0.0183	0.0067	94595	A	G	54.10526
12	rs13201129	1.50E-20	6	0.0071	0.0068	94595	C	T	86.3605
13	rs13330176	9.00E-09	16	0.0046	0.0082	94595	A	T	33.04637
14	rs151719	4.80E-38	6	0.0099	0.0047	94595	T	C	166.282
15	rs1547669	1.60E-27	6	-0.0059	0.0052	94595	G	A	118.1593
16	rs154973	2.70E-34	6	-0.0143	0.0112	94595	T	C	149.1201
17	rs154977	5.60E-64	6	4.00E-04	0.0049	94595	G	C	285.1741
18	rs1571878	4.90E-15	6	-0.0134	0.0053	94595	T	C	61.30078
19	rs1611627	1.30E-21	6	0.0068	0.0057	94595	C	T	91.19817
20	rs17264332	7.10E-19	6	-0.0019	0.0046	94595	G	A	78.73552
21	rs17576984	2.80E-60	6	0.0168	0.0062	94595	T	C	268.1999
22	rs17615220	1.40E-32	6	4.00E-04	0.0073	94595	G	A	141.2764
23	rs1858037	5.90E-10	2	0.0029	0.0054	94595	A	T	38.35468
24	rs187764	4.00E-36	6	-0.0055	0.0119	94595	T	C	157.4902
25	rs1893592	9.80E-09	21	0.0013	0.006	94595	C	A	32.8806
26	rs2050189	7.40E-25	6	-0.0202	0.0048	94595	C	T	105.9938
27	rs206776	3.30E-42	6	0.0049	0.0057	94595	C	T	185.3448
28	rs2107202	3.40E-26	6	0.0059	0.0045	94595	C	T	112.0987
29	rs211453	2.20E-32	6	-0.0017	0.004	94595	G	T	140.3784
30	rs2233434	3.30E-08	6	-0.0135	0.0114	94595	G	A	30.52262
31	rs2239800	2.50E-23	6	0.0108	0.0091	94595	G	A	99.02027
32	rs2244579	1.10E-28	6	0.0147	0.0061	94595	C	G	123.4708
33	rs2251396	1.60E-77	6	-0.0181	0.0056	94595	A	G	347.3527
34	rs2278442	2.30E-08	19	-0.0063	0.0063	94595	A	G	31.22328
35	rs241424	1.60E-16	6	-0.0052	0.0038	94595	A	G	68.04238
36	rs2442728	2.60E-09	6	-0.0171	0.0055	94595	T	G	35.46278
37	rs2451258	6.60E-10	6	0.0061	0.0055	94595	T	C	38.13561
38	rs2476601	1.60E-149	1	0.0048	0.0059	94595	G	A	678.2551
39	rs2561477	5.20E-10	5	-0.0088	0.0055	94595	A	G	38.60115
40	rs2844463	9.50E-40	6	0.0117	0.006	94595	A	G	174.0818
41	rs29232	1.20E-16	6	0.0053	0.0042	94595	T	C	68.60964
42	rs3087243	9.20E-20	2	6.00E-04	0.0037	94595	A	G	82.77339
43	rs3094165	5.80E-21	6	2.00E-04	0.004	94595	G	A	88.23906
44	rs3095150	5.70E-29	6	0.004	0.0042	94595	T	C	124.7761
45	rs3128947	2.00E-115	6	0.0158	0.0085	94595	G	A	521.4956
46	rs3132453	1.10E-15	6	0.0247	0.0073	94595	G	T	64.24291
47	rs3219184	6.40E-10	6	0.0376	0.009	94595	G	A	38.19527
48	rs3749946	4.00E-23	6	0.0195	0.008	94595	A	C	98.08875
49	rs3763326	2.40E-44	6	-0.0135	0.016	94595	C	T	195.1411
50	rs3819721	1.00E-200	6	-2.00E-04	0.0043	94595	A	G	1106.62
51	rs3873444	6.10E-103	6	0.0031	0.0068	94595	T	C	464.1208
52	rs3892710	1.90E-23	6	0.0034	0.0048	94595	T	C	99.56292
53	rs391755	1.50E-50	6	0.0093	0.0124	94595	G	A	223.5779
54	rs4452313	2.70E-10	3	-0.0092	0.0058	94595	T	A	39.88087
55	rs4673266	4.50E-11	2	0.011	0.0061	94595	T	A	43.38373
56	rs4711363	8.90E-15	6	0.0046	0.0058	94595	G	A	60.12521
57	rs4839324	6.50E-13	1	0.0063	0.0073	94595	A	G	51.69013
58	rs4936059	4.60E-08	11	0.009	0.0058	94595	G	A	29.87841
59	rs4959027	6.60E-95	6	0.013	0.0065	94595	G	A	427.2029
60	rs588997	2.20E-30	6	0.0068	0.0041	94595	A	G	131.2357
61	rs624988	4.60E-08	1	0.0022	0.0054	94595	C	T	29.87843
62	rs706778	7.10E-12	10	-0.0062	0.0052	94595	T	C	46.99982
63	rs773566	4.80E-12	1	0.0017	0.0065	94595	C	T	47.76704
64	rs8026898	2.40E-17	15	0.0021	0.0058	94595	A	G	71.7845
65	rs8032939	2.40E-12	15	0.0095	0.0061	94595	C	T	49.1263
66	rs8192585	3.50E-52	6	-5.00E-04	0.0091	94595	A	G	231.0606
67	rs86715	1.10E-13	6	-2.00E-04	0.0085	94595	G	A	55.17983
68	rs872661	1.30E-08	1	0.0057	0.0063	94595	G	A	32.33164
69	rs915654	1.60E-79	6	0.0085	0.004	94595	A	T	356.5348
70	rs9268000	1.00E-200	6	0.005	0.0054	94595	C	A	1143.796
71	rs9275698	1.50E-139	6	-0.0013	0.004	94595	G	A	632.4024
72	rs9276831	8.40E-72	6	0.0022	0.0061	94595	G	A	321.0858
73	rs9276931	3.10E-33	6	-0.0107	0.0067	94595	G	A	144.271
74	rs9295939	1.60E-16	6	-0.003	0.0162	94595	G	A	68.04276
75	rs9366829	1.30E-09	6	0.0027	0.0036	94595	G	A	36.81354
76	rs9368744	5.80E-10	6	-0.0342	0.0321	94595	T	C	38.38768
77	rs9501626	3.10E-97	6	0.0021	0.0062	94595	A	C	437.9002
78	rs9603608	7.60E-11	13	-0.0031	0.0054	94595	C	A	42.35802
79	rs9653442	3.60E-12	2	0.0013	0.0052	94595	T	C	48.33155

Table S6 The information of SNPs selected for MR analysis between RA and HDL

Number	SNPs	*P*-value	Chromosome	Beta	SE	Sample size	EA	OA	*F*-value
1	rs1002985	3.40E-09	6	0.0096	0.0083	94595	T	C	34.94035
2	rs10985070	1.70E-08	9	-0.0033	0.0034	94595	A	C	31.81034
3	rs11102712	1.00E-28	1	-0.0027	0.0078	94595	T	C	123.6595
4	rs11574914	1.50E-13	9	-0.008	0.0036	94595	A	G	54.57048
5	rs11751928	7.60E-19	6	-0.0023	0.0066	94595	T	C	78.60156
6	rs12204421	1.60E-26	6	0.0012	0.0056	94595	G	A	113.5924
7	rs12232497	3.60E-09	17	0.0249	0.0048	94595	C	T	34.82902
8	rs1265093	1.20E-62	6	-0.0014	0.0039	94595	A	G	279.0669
9	rs1265095	8.70E-13	6	7.00E-04	0.0049	94595	A	G	51.11767
10	rs12665140	1.60E-13	6	0.0014	0.0159	94595	A	C	54.44349
11	rs12764378	1.90E-13	10	-0.0097	0.0062	94595	A	G	54.10526
12	rs13201129	1.50E-20	6	0.0036	0.0063	94595	C	T	86.3605
13	rs13330176	9.00E-09	16	0.0077	0.0074	94595	A	T	33.04637
14	rs151719	4.80E-38	6	0.0021	0.0044	94595	T	C	166.282
15	rs1547669	1.60E-27	6	0.0043	0.0048	94595	G	A	118.1593
16	rs154973	2.70E-34	6	0.0176	0.0102	94595	T	C	149.1201
17	rs154977	5.60E-64	6	0.0163	0.0045	94595	G	C	285.1741
18	rs1571878	4.90E-15	6	0.005	0.0049	94595	T	C	61.30078
19	rs1611627	1.30E-21	6	2.00E-04	0.0052	94595	C	T	91.19817
20	rs17264332	7.10E-19	6	-0.0023	0.0043	94595	G	A	78.73552
21	rs17576984	2.80E-60	6	-0.0198	0.0058	94595	T	C	268.1999
22	rs17615220	1.40E-32	6	-0.0089	0.0069	94595	G	A	141.2764
23	rs1858037	5.90E-10	2	0.0012	0.005	94595	A	T	38.35468
24	rs187764	4.00E-36	6	0.0121	0.0106	94595	T	C	157.4902
25	rs1893592	9.80E-09	21	-0.0054	0.0056	94595	C	A	32.8806
26	rs2050189	7.40E-25	6	-0.0073	0.0044	94595	C	T	105.9938
27	rs206776	3.30E-42	6	0.0062	0.0052	94595	C	T	185.3448
28	rs2107202	3.40E-26	6	0.0011	0.0042	94595	C	T	112.0987
29	rs211453	2.20E-32	6	0.0062	0.0037	94595	G	T	140.3784
30	rs2233434	3.30E-08	6	-0.0163	0.0105	94595	G	A	30.52262
31	rs2239800	2.50E-23	6	0.0138	0.0084	94595	G	A	99.02027
32	rs2244579	1.10E-28	6	0.002	0.0056	94595	C	G	123.4708
33	rs2251396	1.60E-77	6	-0.0178	0.0052	94595	A	G	347.3527
34	rs2278442	2.30E-08	19	7.00E-04	0.0058	94595	A	G	31.22328
35	rs241424	1.60E-16	6	0.0019	0.0036	94595	A	G	68.04238
36	rs2442728	2.60E-09	6	-0.0038	0.0051	94595	T	G	35.46278
37	rs2451258	6.60E-10	6	0.0038	0.0051	94595	T	C	38.13561
38	rs2476601	1.60E-149	1	-0.0205	0.0055	94595	G	A	678.2551
39	rs2561477	5.20E-10	5	-0.0026	0.005	94595	A	G	38.60115
40	rs2844463	9.50E-40	6	-0.0154	0.0056	94595	A	G	174.0818
41	rs29232	1.20E-16	6	0.0057	0.0039	94595	T	C	68.60964
42	rs3087243	9.20E-20	2	-0.0059	0.0035	94595	A	G	82.77339
43	rs3094165	5.80E-21	6	0.0026	0.0038	94595	G	A	88.23906
44	rs3095150	5.70E-29	6	-0.0051	0.0039	94595	T	C	124.7761
45	rs3128947	2.00E-115	6	0.0014	0.0075	94595	G	A	521.4956
46	rs3132453	1.10E-15	6	-0.006	0.0069	94595	G	T	64.24291
47	rs3219184	6.40E-10	6	0.0179	0.0084	94595	G	A	38.19527
48	rs3749946	4.00E-23	6	-0.0271	0.0074	94595	A	C	98.08875
49	rs3763326	2.40E-44	6	0.0017	0.015	94595	C	T	195.1411
50	rs3819721	1.00E-200	6	0.0101	0.0041	94595	A	G	1106.62
51	rs3873444	6.10E-103	6	-0.004	0.0064	94595	T	C	464.1208
52	rs3892710	1.90E-23	6	-0.0063	0.0045	94595	T	C	99.56292
53	rs391755	1.50E-50	6	-0.0318	0.0112	94595	G	A	223.5779
54	rs4452313	2.70E-10	3	2.00E-04	0.0053	94595	T	A	39.88087
55	rs4673266	4.50E-11	2	-0.0019	0.0056	94595	T	A	43.38373
56	rs4711363	8.90E-15	6	0.0025	0.0054	94595	G	A	60.12521
57	rs4839324	6.50E-13	1	0.0027	0.0067	94595	A	G	51.69013
58	rs4936059	4.60E-08	11	0.0047	0.0054	94595	G	A	29.87841
59	rs4959027	6.60E-95	6	-0.0088	0.0059	94595	G	A	427.2029
60	rs588997	2.20E-30	6	5.00E-04	0.0038	94595	A	G	131.2357
61	rs624988	4.60E-08	1	-0.0025	0.005	94595	C	T	29.87843
62	rs706778	7.10E-12	10	0.0067	0.0048	94595	T	C	46.99982
63	rs773566	4.80E-12	1	-2.00E-04	0.006	94595	C	T	47.76704
64	rs8026898	2.40E-17	15	0.0013	0.0054	94595	A	G	71.7845
65	rs8032939	2.40E-12	15	-0.0069	0.0056	94595	C	T	49.1263
66	rs8192585	3.50E-52	6	-0.0098	0.0085	94595	A	G	231.0606
67	rs86715	1.10E-13	6	0.0031	0.0079	94595	G	A	55.17983
68	rs872661	1.30E-08	1	-0.0012	0.0057	94595	G	A	32.33164
69	rs915654	1.60E-79	6	0.0033	0.0038	94595	A	T	356.5348
70	rs9268000	1.00E-200	6	-0.0047	0.005	94595	C	A	1143.796
71	rs9275698	1.50E-139	6	-0.0059	0.0037	94595	G	A	632.4024
72	rs9276831	8.40E-72	6	6.00E-04	0.0057	94595	G	A	321.0858
73	rs9276931	3.10E-33	6	-0.0188	0.0063	94595	G	A	144.271
74	rs9295939	1.60E-16	6	0.0112	0.0152	94595	G	A	68.04276
75	rs9366829	1.30E-09	6	-8.00E-04	0.0034	94595	G	A	36.81354
76	rs9368744	5.80E-10	6	0.0359	0.03	94595	T	C	38.38768
77	rs9501626	3.10E-97	6	0.0065	0.0058	94595	A	C	437.9002
78	rs9603608	7.60E-11	13	-0.0018	0.005	94595	C	A	42.35802
79	rs9653442	3.60E-12	2	0.0063	0.0048	94595	T	C	48.33155

Table S7 The information of SNPs selected for MR analysis between SLE and TC

Number	SNPs	*P* -value	Chromosome	Beta	SE	Sample size	EA	OA	*F* -value
1	rs10200680	4.96E-09	2	-0.0016	0.0098	94595	T	C	34.20392
2	rs1078324	7.11E-20	5	0.021	0.0177	94595	A	C	83.28446
3	rs12662632	5.53E-11	6	-0.0073	0.0144	94595	C	G	42.98038
4	rs13019891	1.65E-83	2	-0.0053	0.0053	94595	T	G	374.8476
5	rs1464446	2.79E-16	3	-0.0057	0.0073	94595	T	G	66.94478
6	rs154977	5.65E-15	6	0.0106	0.0047	94595	G	C	61.01911
7	rs17615220	2.86E-12	6	-0.0078	0.0071	94595	G	A	48.78294
8	rs185819	2.32E-37	6	0.0059	0.0036	94595	C	T	163.1505
9	rs1894406	2.22E-20	6	0.0183	0.0038	94595	T	C	85.58207
10	rs194675	2.71E-12	6	0.0116	0.0054	94595	A	T	48.8908
11	rs2022082	2.78E-12	6	0.0061	0.0057	94595	C	T	48.83822
12	rs2076530	2.13E-23	6	0.0216	0.0051	94595	C	T	99.33799
13	rs2293765	3.61E-19	2	0.014	0.0052	94595	A	C	80.07252
14	rs2293861	9.84E-21	6	0.0215	0.0068	94595	T	C	87.19266
15	rs2294473	3.89E-08	6	0.0028	0.0036	94595	C	G	30.20593
16	rs2395004	3.98E-08	6	-0.0156	0.0051	94595	C	G	30.16009
17	rs2431697	2.60E-14	5	0.0088	0.0053	94595	C	T	58.01522
18	rs2442728	4.63E-24	6	-0.0232	0.0054	94595	T	G	102.3588
19	rs2517554	1.43E-10	6	-0.0059	0.0056	94595	T	C	41.11707
20	rs2573219	1.13E-42	2	0.0055	0.0087	94595	C	A	187.4711
21	rs2853950	1.71E-13	6	0.0222	0.0053	94595	T	C	54.30977
22	rs3095339	9.05E-34	6	-0.0184	0.006	94595	G	A	146.7157
23	rs3131932	3.03E-18	6	0.0105	0.0053	94595	A	G	75.86964
24	rs353608	2.93E-11	11	-0.0062	0.0051	94595	G	A	44.22171
25	rs3819720	2.10E-15	6	-0.0089	0.0057	94595	A	G	62.96659
26	rs389884	2.92E-102	6	-0.0481	0.0084	94595	G	A	460.9908
27	rs4585609	1.57E-10	6	0.0105	0.0043	94595	C	T	40.93853
28	rs4661543	9.40E-11	1	-0.0058	0.0087	94595	G	T	41.94263
29	rs4728142	5.37E-28	7	-0.0082	0.0056	94595	A	G	120.3265
30	rs4916215	5.07E-11	1	0.0058	0.0058	94595	T	C	43.15163
31	rs512681	8.75E-13	2	0.0089	0.0073	94595	A	C	51.10645
32	rs572708	1.49E-09	6	0.0083	0.0076	94595	C	T	36.54206
33	rs6671847	6.64E-12	1	-0.023	0.0052	94595	A	G	47.13087
34	rs6679677	4.55E-13	1	-0.0083	0.0057	94595	A	C	52.39197
35	rs670369	6.83E-09	6	0.0216	0.0068	94595	T	C	33.58255
36	rs6889239	2.19E-18	5	-4.00E-04	0.0058	94595	C	T	76.51123
37	rs6903160	9.27E-09	6	-0.0051	0.0054	94595	A	T	32.9883
38	rs7097397	8.60E-11	10	0.0065	0.0052	94595	A	G	42.11577
39	rs721803	1.81E-09	6	-0.0041	0.004	94595	A	G	36.16794
40	rs7386188	1.10E-08	8	-0.0161	0.0142	94595	G	T	32.6477
41	rs7601754	9.31E-25	2	-0.0093	0.0067	94595	A	G	105.5375
42	rs7746553	1.16E-11	6	0.021	0.005	94595	G	C	46.04375
43	rs7823055	1.64E-34	8	0.0031	0.0061	94595	T	G	150.1072
44	rs885952	8.27E-22	6	0.0068	0.0052	94595	T	C	92.09312
45	rs9266791	3.53E-26	6	0.0235	0.0046	94595	C	T	112.0218
46	rs9461633	7.98E-11	6	0.0217	0.0062	94595	G	A	42.26264
47	rs946173	2.69E-11	1	-0.0015	0.0086	94595	G	A	44.38952
48	rs9468344	4.00E-13	6	-0.0153	0.0059	94595	G	T	52.64146
49	rs9852014	2.26E-36	3	0.0041	0.0103	94595	G	A	158.6276

Table S8 The information of SNPs selected for MR analysis between SLE and LDL

Number	SNPs	*P* -value	Chromosome	Beta	SE	Sample size	EA	OA	*F* -value
1	rs10200680	4.96E-09	2	-0.014	0.01	94595	T	C	34.2039198
2	rs1078324	7.11E-20	5	0.0118	0.018	94595	A	C	83.2844647
3	rs12662632	5.53E-11	6	-0.0129	0.0149	94595	C	G	42.9803809
4	rs13019891	1.65E-83	2	-0.0061	0.0054	94595	T	G	374.847615
5	rs1464446	2.79E-16	3	-0.0095	0.0075	94595	T	G	66.9447794
6	rs154977	5.65E-15	6	4.00E-04	0.0049	94595	G	C	61.019111
7	rs17615220	2.86E-12	6	4.00E-04	0.0073	94595	G	A	48.7829382
8	rs185819	2.32E-37	6	6.00E-04	0.0038	94595	C	T	163.15048
9	rs1894406	2.22E-20	6	0.0172	0.0039	94595	T	C	85.5820736
10	rs194675	2.71E-12	6	0.0103	0.0056	94595	A	T	48.8907984
11	rs2022082	2.78E-12	6	0.0033	0.0058	94595	C	T	48.83822
12	rs2076530	2.13E-23	6	0.0087	0.0052	94595	C	T	99.3379872
13	rs2293765	3.61E-19	2	0.012	0.0053	94595	A	C	80.0725239
14	rs2293861	9.84E-21	6	0.0179	0.007	94595	T	C	87.1926638
15	rs2294473	3.89E-08	6	-0.0014	0.0037	94595	C	G	30.2059267
16	rs2395004	3.98E-08	6	-0.0134	0.0053	94595	C	G	30.1600891
17	rs2431697	2.60E-14	5	0.0103	0.0054	94595	C	T	58.0152161
18	rs2442728	4.63E-24	6	-0.0171	0.0055	94595	T	G	102.358781
19	rs2517554	1.43E-10	6	0.0044	0.0057	94595	T	C	41.1170713
20	rs2573219	1.13E-42	2	0.0023	0.0089	94595	C	A	187.471115
21	rs2853950	1.71E-13	6	0.0187	0.0054	94595	T	C	54.3097698
22	rs3095339	9.05E-34	6	-0.0115	0.0061	94595	G	A	146.71572
23	rs3131932	3.03E-18	6	0.01	0.0054	94595	A	G	75.8696437
24	rs353608	2.93E-11	11	-0.0051	0.0052	94595	G	A	44.2217073
25	rs3819720	2.10E-15	6	-0.0036	0.0058	94595	A	G	62.9665878
26	rs389884	2.92E-102	6	-0.0235	0.0086	94595	G	A	460.990804
27	rs4585609	1.57E-10	6	0.0055	0.0044	94595	C	T	40.9385265
28	rs4661543	9.40E-11	1	-0.0015	0.0089	94595	G	T	41.9426296
29	rs4728142	5.37E-28	7	-0.0061	0.0057	94595	A	G	120.326529
30	rs4916215	5.07E-11	1	0.0076	0.0059	94595	T	C	43.1516327
31	rs512681	8.75E-13	2	0.0127	0.0075	94595	A	C	51.1064491
32	rs572708	1.49E-09	6	-0.0019	0.0078	94595	C	T	36.5420581
33	rs6671847	6.64E-12	1	-0.0245	0.0054	94595	A	G	47.1308741
34	rs6679677	4.55E-13	1	-0.0051	0.006	94595	A	C	52.3919683
35	rs670369	6.83E-09	6	0.0232	0.0069	94595	T	C	33.5825457
36	rs6889239	2.19E-18	5	-0.002	0.0059	94595	C	T	76.5112326
37	rs6903160	9.27E-09	6	0.0021	0.0055	94595	A	T	32.9882999
38	rs7097397	8.60E-11	10	0.0075	0.0053	94595	A	G	42.1157683
39	rs721803	1.81E-09	6	-0.0064	0.0042	94595	A	G	36.1679387
40	rs7386188	1.10E-08	8	-0.0155	0.0145	94595	G	T	32.6477016
41	rs7601754	9.31E-25	2	-0.0048	0.0068	94595	A	G	105.537463
42	rs7746553	1.16E-11	6	0.0149	0.0052	94595	G	C	46.0437492
43	rs7823055	1.64E-34	8	0.003	0.0062	94595	T	G	150.107188
44	rs885952	8.27E-22	6	0.006	0.0053	94595	T	C	92.0931153
45	rs9266791	3.53E-26	6	0.0151	0.0048	94595	C	T	112.021805
46	rs9461633	7.98E-11	6	0.0153	0.0063	94595	G	A	42.262639
47	rs946173	2.69E-11	1	-8.00E-04	0.0089	94595	G	A	44.3895215
48	rs9468344	4.00E-13	6	-0.0075	0.006	94595	G	T	52.6414626
49	rs9852014	2.26E-36	3	-0.006	0.0105	94595	G	A	158.627555

Table S9 The information of SNPs selected for MR analysis between SLE and HDL

Number	SNPs	*P* -value	Chromosome	Beta	SE	Sample size	EA	OA	*F* -value
1	rs10200680	4.96E-09	2	-0.0091	0.0093	94595	T	C	34.20392
2	rs1078324	7.11E-20	5	0.0049	0.016	94595	A	C	83.28446
3	rs12662632	5.53E-11	6	0.0169	0.014	94595	C	G	42.98038
4	rs13019891	1.65E-83	2	-2.00E-04	0.0049	94595	T	G	374.8476
5	rs1464446	2.79E-16	3	-0.0018	0.0068	94595	T	G	66.94478
6	rs154977	5.65E-15	6	0.0163	0.0045	94595	G	C	61.01911
7	rs17615220	2.86E-12	6	-0.0089	0.0069	94595	G	A	48.78294
8	rs185819	2.32E-37	6	0.0111	0.0035	94595	C	T	163.1505
9	rs1894406	2.22E-20	6	0.0073	0.0036	94595	T	C	85.58207
10	rs194675	2.71E-12	6	-0.0011	0.0052	94595	A	T	48.8908
11	rs2022082	2.78E-12	6	0.0043	0.0054	94595	C	T	48.83822
12	rs2076530	2.13E-23	6	0.0108	0.0048	94595	C	T	99.33799
13	rs2293765	3.61E-19	2	0.0014	0.0049	94595	A	C	80.07252
14	rs2293861	9.84E-21	6	-0.0021	0.0065	94595	T	C	87.19266
15	rs2294473	3.89E-08	6	0.0039	0.0035	94595	C	G	30.20593
16	rs2395004	3.98E-08	6	-0.0095	0.0048	94595	C	G	30.16009
17	rs2431697	2.60E-14	5	-0.0024	0.0051	94595	C	T	58.01522
18	rs2442728	4.63E-24	6	-0.0038	0.0051	94595	T	G	102.3588
19	rs2517554	1.43E-10	6	-0.0154	0.0053	94595	T	C	41.11707
20	rs2573219	1.13E-42	2	-0.006	0.0082	94595	C	A	187.4711
21	rs2853950	1.71E-13	6	0.0016	0.005	94595	T	C	54.30977
22	rs3095339	9.05E-34	6	-0.0084	0.0056	94595	G	A	146.7157
23	rs3131932	3.03E-18	6	-0.001	0.0049	94595	A	G	75.86964
24	rs353608	2.93E-11	11	0.0012	0.0048	94595	G	A	44.22171
25	rs3819720	2.10E-15	6	-0.008	0.0053	94595	A	G	62.96659
26	rs389884	2.92E-102	6	-0.0245	0.0077	94595	G	A	460.9908
27	rs4585609	1.57E-10	6	0.0118	0.0041	94595	C	T	40.93853
28	rs4661543	9.40E-11	1	0.0077	0.0083	94595	G	T	41.94263
29	rs4728142	5.37E-28	7	0.0026	0.0053	94595	A	G	120.3265
30	rs4916215	5.07E-11	1	-0.0051	0.0054	94595	T	C	43.15163
31	rs512681	8.75E-13	2	-0.006	0.0069	94595	A	C	51.10645
32	rs572708	1.49E-09	6	0.0108	0.0071	94595	C	T	36.54206
33	rs6671847	6.64E-12	1	-0.0026	0.005	94595	A	G	47.13087
34	rs6679677	4.55E-13	1	0.0204	0.0056	94595	A	C	52.39197
35	rs670369	6.83E-09	6	-0.0053	0.0064	94595	T	C	33.58255
36	rs6889239	2.19E-18	5	0.0017	0.0055	94595	C	T	76.51123
37	rs6903160	9.27E-09	6	-0.0028	0.005	94595	A	T	32.9883
38	rs7097397	8.60E-11	10	0.0037	0.0049	94595	A	G	42.11577
39	rs721803	1.81E-09	6	0.0048	0.0039	94595	A	G	36.16794
40	rs7386188	1.10E-08	8	-0.0089	0.0133	94595	G	T	32.6477
41	rs7601754	9.31E-25	2	-0.0016	0.0063	94595	A	G	105.5375
42	rs7746553	1.16E-11	6	0.0049	0.0048	94595	G	C	46.04375
43	rs7823055	1.64E-34	8	-0.0105	0.0057	94595	T	G	150.1072
44	rs885952	8.27E-22	6	-0.0064	0.0048	94595	T	C	92.09312
45	rs9266791	3.53E-26	6	0.0159	0.0044	94595	C	T	112.0218
46	rs9461633	7.98E-11	6	0.0069	0.0058	94595	G	A	42.26264
47	rs946173	2.69E-11	1	0.0063	0.0082	94595	G	A	44.38952
48	rs9468344	4.00E-13	6	-0.0109	0.0055	94595	G	T	52.64146
49	rs9852014	2.26E-36	3	0.0025	0.0096	94595	G	A	158.6276
