# Supplementary material for: Causal relationships between rheumatism and dyslipidemia: A two-sample Mendelian randomization study
Source: Front Endocrinol (Lausanne). 2022 Aug 31;13:961505. doi: 10.3389/fendo.2022.961505 (PMC9470850; doi:10.3389/fendo.2022.961505)
Supplement: Supplementary file 3 [file DataSheet_3.docx]

**Table S10 The Mendelian randomization (MR) analysis results with regard to**

**causal effect of RA on TC, LDL and HDL levels**

| Outcome | Method | SNP(n) | Beta | 95%CI | *P*-value |
| --- | --- | --- | --- | --- | --- |
| TC | Weighted median | 78 | 0.008 | 4.86×10^-4^, 0.017 | 0.064 |
|  | Inverse variance weighted | 78 | 0.004 | -0.005, 0.014 | 0.386 |
|  | MR Egger | 78 | -0.003 | -0.009, 0.014 | 0.764 |
| LDL | Weighted median | 79 | 6.4×10^-4^ | -0.008, 0.007 | 0.871 |
|  | Inverse variance weighted | 79 | 0.002 | -0.006, 0.009 | 0.635 |
|  | MR Egger | 79 | -0.004 | -0.017, 0.009 | 0.547 |
| HDL | Weighted median | 79 | 0.005 | -0.003, 0.013 | 0.200 |
|  | Inverse variance weighted | 79 | 0.006 | -7.5×10^-4^, 0.013 | 0.081 |
|  | MR Egger | 79 | 0.009 | -0.002, 0.021 | 0.124 |

Beta: a ratio of changes in standard deviations; RA: rheumatoid arthritis; TC: total cholesterol; LDL: low density lipoprotein; HDL: high density lipoprotein; SNP: single nucleotide polymorphism; CI: confidence interval.
